# *Schistosoma mansoni* vaccine candidates identified by unbiased phage display screening in self-cured rhesus macaques

**DOI:** 10.1038/s41541-023-00803-x

**Published:** 2024-01-04

**Authors:** Daisy Woellner-Santos, Ana C. Tahira, João V. M. Malvezzi, Vinicius Mesel, David A. Morales-Vicente, Monalisa M. Trentini, Lázaro M. Marques-Neto, Isaac A. Matos, Alex I. Kanno, Adriana S. A. Pereira, André A. R. Teixeira, Ricardo J. Giordano, Luciana C. C. Leite, Carlos A. B. Pereira, Ricardo DeMarco, Murilo S. Amaral, Sergio Verjovski-Almeida

**Affiliations:** 1 Laboratório de Ciclo Celular, Instituto Butantan, São Paulo, SP Brazil; 2 Instituto de Química, Universidade de São Paulo, São Paulo, SP Brazil; 3 Instituto de Matemática e Estatística, Universidade de São Paulo, São Paulo, SP Brazil; 4 Laboratório de Desenvolvimento de Vacinas, Instituto Butantan, São Paulo, SP Brazil; 5 Instituto de Física de São Carlos, Universidade de São Paulo, São Carlos, SP Brazil; 6 Present Address: Institute for Protein Innovation, Boston, MA USA

**Keywords:** Parasitic infection, Peptide vaccines

## Abstract

Schistosomiasis, a challenging neglected tropical disease, affects millions of people worldwide. Developing a prophylactic vaccine against *Schistosoma mansoni* has been hindered by the parasite’s biological complexity. In this study, we utilized the innovative phage-display immunoprecipitation followed by a sequencing approach (PhIP-Seq) to screen the immune response of 10 infected rhesus macaques during self-cure and challenge-resistant phases, identifying vaccine candidates. Our high-throughput *S. mansoni* synthetic DNA phage-display library encoded 99.6% of 119,747 58-mer peptides, providing comprehensive coverage of the parasite’s proteome. Library screening with rhesus macaques’ antibodies, from the early phase of establishment of parasite infection, identified significantly enriched epitopes of parasite extracellular proteins known to be expressed in the digestive tract, shifting towards intracellular proteins during the late phase of parasite clearance. Immunization of mice with a selected pool of PhIP-Seq-enriched phage-displayed peptides from MEG proteins, cathepsins B, and asparaginyl endopeptidase significantly reduced worm burden in a vaccination assay. These findings enhance our understanding of parasite-host immune responses and provide promising prospects for developing an effective schistosomiasis vaccine.

## Introduction

Schistosomiasis is a neglected tropical disease that poses a significant public health threat worldwide, particularly in low- and middle-income countries^1^. Despite four decades of control strategies, this disease afflicts over 600 million people in 78 countries with substantial morbidity and mortality^1,2^. While chemotherapy with praziquantel is the primary treatment for schistosomiasis, there are concerns about drug resistance and the limited availability and accessibility of treatment in many affected areas^3^. Developing a vaccine would be a game-changer in mitigating the sorrow of millions of people and effectively eliminating and controlling schistosomiasis^4^.

Vaccine-promising strategies include radiation-attenuated cercariae and recombinant subunit vaccines^5–7^. Although the complexity of orchestrating a protective immune response against this large multicellular organism, irradiated cercariae immunization opened a window for optimism, reaching high levels of partial protection in mice^5,8^ and primates^9,10^. Subunit vaccines have been used to test over one hundred potential antigens^6^, such as Sm-p80, Sm-TSP-2, and Sm14^11^. While Sm-p80 conferred partial protection in mice and non-human primates^12–14^, Sm-TSP-2 demonstrated efficacy in mice^15^ and has been shown to be safe in a Phase 1 trial^16^ and in a Phase 1b clinical trial in a region of Brazil with ongoing *S. mansoni* transmission, eliciting significant IgG responses against the vaccine antigen^17^. Protein Sm14 did protect vaccinated mice against *S. mansoni*^18^, and Sm14 vaccination in a Phase 1a trial of adult, male volunteers^19^ and in a Phase1b trial of adult females^20^ from a non-endemic area for schistosomiasis showed tolerability and specific IgG immune responses. Despite promising preclinical results, most recombinant protein-based vaccines fail at different clinical stages to elicit a robust immune response or protection, as reviewed by Panzner et al. ^11^; this was the case of *S. haematobium* Sh28GST/Alhydrogel (Bilharvax), which remains as the only vaccine candidate to complete clinical phase III trial, in which a sufficient efficacy was not reached^21^.

To address this challenge, we employed rhesus macaques (*Macaca mulatta*) as a non-permissive host for *S. mansoni* infection, a unique and valuable model for studying the immune response and development of vaccines against schistosomiasis^22–24^. In a previous study, we found that rhesus macaques infected and challenged with *S. mansoni* (see Methods) did reduce the longevity of adult worms in primary infections and developed robust resistance to a challenge^23^. These processes were attributed to the disruption of the parasite homeostasis and nutrient uptake through antibody-mediated mechanisms^23^.

**Fig. 1:**
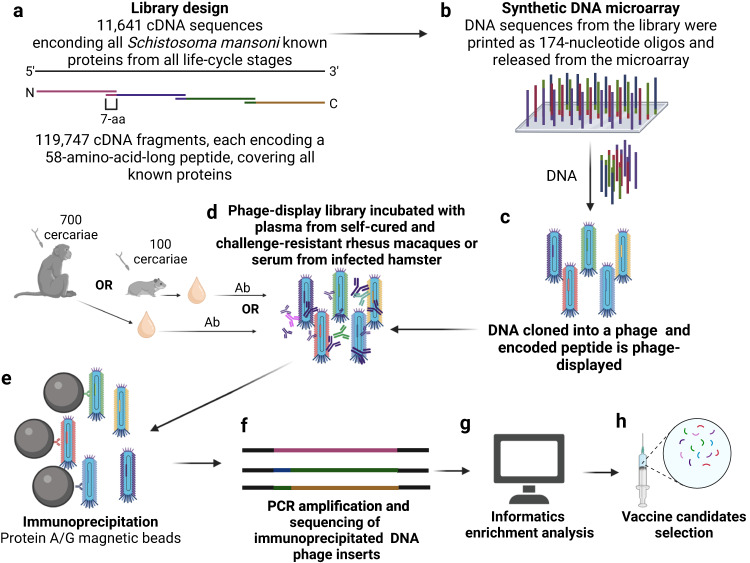
Phage Immunoprecipitation-Sequencing (PhIP-Seq) approach for screening of *S. mansoni* vaccine candidates. **a** A phage-display library was constructed encoding 11,641 known proteins from all *S. mansoni* life-cycle stages. The library consisted of 119,747 complementary Deoxyribonucleic Acid (cDNA) sequences, each encoding a 58-amino-acid-long peptide with a 7-amino-acid (7-aa) overlap between consecutive sequences. **b** DNA sequences with 174-nucleotides were synthesized and released on a DNA-releasable microarray. **c** These sequences were cloned, and each encoded peptide was expressed as a fusion with the major capsid protein (pVIII) of an M13 bacteriophage display vector. **d** The library was incubated with antibodies (Ab) in the plasma from rhesus macaques of a previous study from our group collected at different time points along the self-cure and resistance to challenge phases^23^ or with serum from hamsters as a permissive control. **e** Phages bound to antibodies were immunoprecipitated with protein A/G magnetic beads, **f** recovered DNA inserts were polymerase-chain-reaction (PCR) amplified, and high-throughput sequenced. **g** Enriched peptides were analyzed with bioinformatics tools (see Methods), and **h** potential vaccine candidates were identified. Created with Biorender.com.

Furthering our understanding of the humoral response against specific targets that impair the parasite’s life cycle within the rhesus macaques blood vessels, we constructed a large-scale synthetic DNA phage-display library, encompassing 58-mer-long peptides, for the first time covering the sequences of all known *S. mansoni* proteins (Fig. 1). This ground-breaking approach addresses the challenges of unbiased screening of the vast *Schistosoma* proteome by combining phage-display technology with high-throughput DNA synthesis^25–27^. This comprehensive peptide library was screened against plasma samples from rhesus macaques collected in a previous study from our group^23^ during the self-cure and challenge-resistant phases. Screening the library against serum samples from infected hamsters (see Methods) was employed as a permissive control^23^. Phages captured by antibodies in the plasma/serum from both infection models were immunoprecipitated (IP), PCR amplified, and deep-sequenced using the recently developed PhIP-Seq technique^25–27^ (Fig. 1).

PhIP-Seq enriched sequences were selected based on macaques’ immune response over time and curated with information on the previously known parasite’s life-stage expression and the known localization at the host-parasite interface. The most promising candidates were tested with a pilot vaccination assay in mice (see Methods) using peptide phage-based immunization^28^. Here, we used a peptide microarray to confirm the PhIP-Seq-identified vaccine candidates and to map and compare the epitopes recognized by rhesus macaques with the antibody responses from permissive controls. Our approach offers valuable insights that could lead to identifying new therapeutic strategies and developing a multi-antigen and effective vaccine against this debilitating disease.

## Results

### A synthetic phage-display library provides unbiased coverage of the *S. mansoni* proteome

**Fig. 2:**
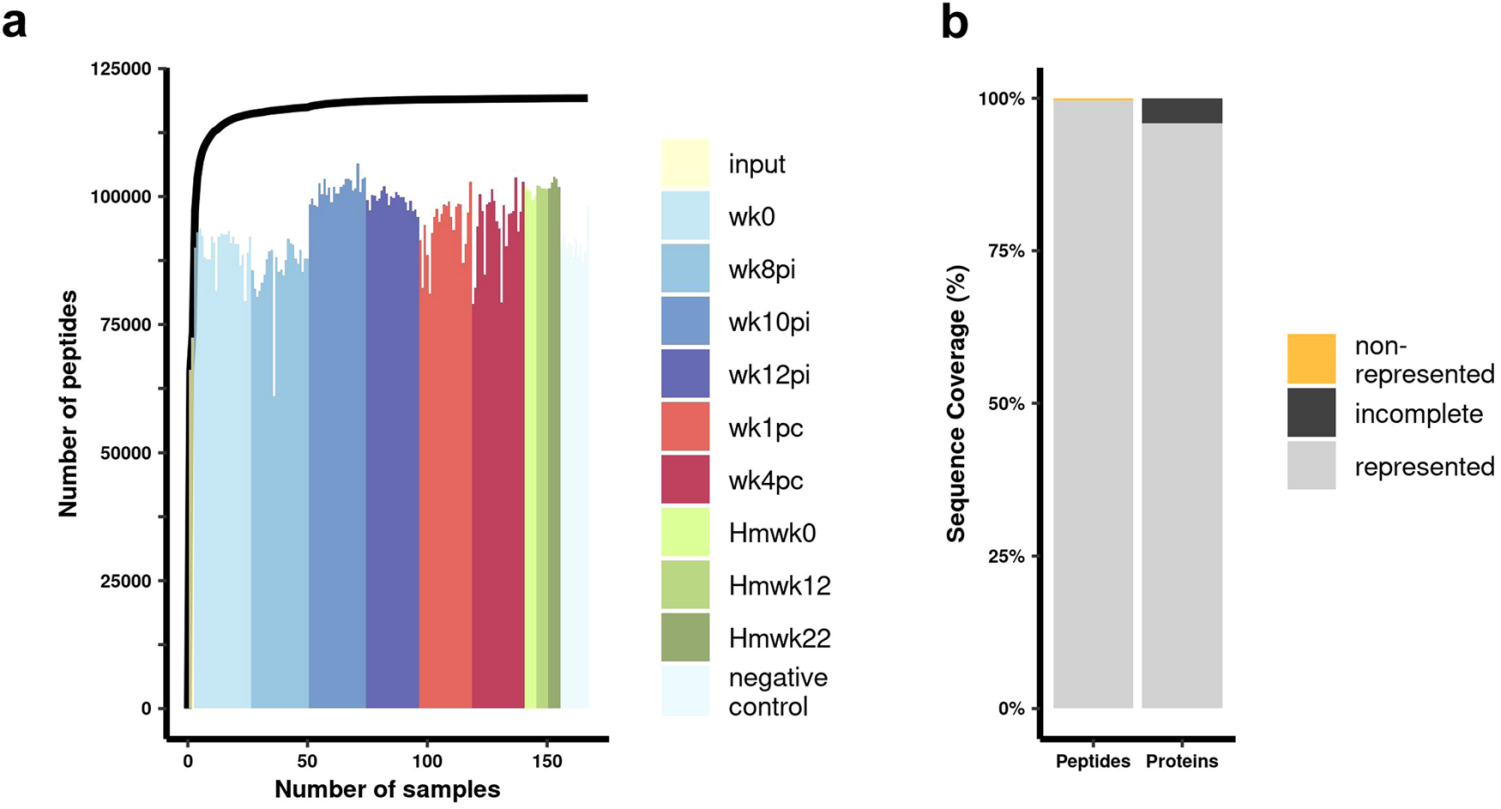
PhIP-Seq provides exceptional coverage of the *S. mansoni* proteome. **a** The histogram illustrates the number of different peptides detected in 167 sequenced samples from a phage-display library encoding 119,747 unique peptides. The samples include two input libraries (input), 138 PhIP-Seq duplicate samples obtained with 12 rhesus macaques’ plasma collected at six different time points, namely before infection (wk0), three time points post-infection (pi) (wk8pi, wk10pi, and wk12pi), as well as at two time points post-challenge (pc) (wk1pc and wk4pc); also, 15 PhIP-Seq samples were obtained at three time points, with five hamsters’ (Hm) sera each, collected at Hmwk0, Hmwk12, and Hmwk22, and 12 PhIP-Seq samples of negative controls (magnetic beads only, no plasma/serum). Different colors represent input libraries (yellow), the six different weeks at which the PhIP-Seq data were collected with rhesus macaques’ plasma (shades of blue and red), or the three PhIP-Seq datasets with hamster serum samples (shades of green), and PhIP-Seq negative controls (light blue; refer to the color legend on the right). The black line graph depicts the cumulative number of different peptides detected when combining sequencing data from the multiple samples. For the line graph, the X-axis represents the number of combined samples, while the Y-axis represents the number of different peptides detected. **b** The bar graph provides an overview of the library coverage based on the sequenced data from all samples, focusing on the representation of peptides (left bar) and proteins (right bar). The Y-axis shows the percentage of represented (gray) or non-represented (orange) peptides (left bar), and the percentage of fully represented (gray) or incomplete (black) proteins (right bar) (see legend on the right side of the graph).

We constructed a comprehensive phage-display library comprising 119,747 sequences encoding 112,396 distinct peptides. Redundant peptide sequences can be distinguished in the sequencing data by different codon usage resulting from separate codon optimization. Remarkably, by combining the oligos sequenced in the input samples with those identified in the immunoprecipitated samples from rhesus macaques’ plasma, hamsters’ serum, and negative controls, a total of 99.6% (119,218/119,747) of the peptides encoded in the synthetic oligonucleotide library were detected (Fig. 2a, b). Supplementary Table 1 (in Supplementary Information) shows the PhIP-seq counts obtained for each peptide at the 167 processed samples.

Out of the total peptides encoded in the synthetic oligonucleotide library, only 0.4% (529 peptides) were not detected in any sequenced sample (Fig. 2b) (see Supplementary Information, Supplementary Table 1). Among these undetected peptides, seven proteins were missing (non-represented), accounting for 0.06% (7/11,641proteins) of the encoded proteome (Fig. 2b).

### Bayesian statistical analysis determined significantly enriched *S. mansoni* peptides captured by rhesus macaques’ antibodies

The PhIP-Seq data were subjected to Bayesian statistical analyses (see Methods) to determine significantly enriched peptides captured by antibodies from 10 rhesus macaques at each sequenced time point: wk0 (before infection), wk8, wk10, and wk12 post-infection, as well as wk1 and wk4 post-challenge (Supplementary Information, Supplementary Table 2). Samples from 2 macaques determined to be outliers^23^, namely Rh1 and Rh10 (see Methods), were excluded from these enrichment analyses. Enrichment results obtained with the Bayesian statistical analysis were compared with the two well-established PhIP-Seq statistical methods described in Supplementary Information. The comparisons revealed that the Bayesian analysis was more suitable for handling group comparison statistics in the present study. In contrast, the other statistical methods proved too restrictive in identifying enriched peptides captured by plasma samples from ten rhesus macaques at each time point.

**Fig. 3:**
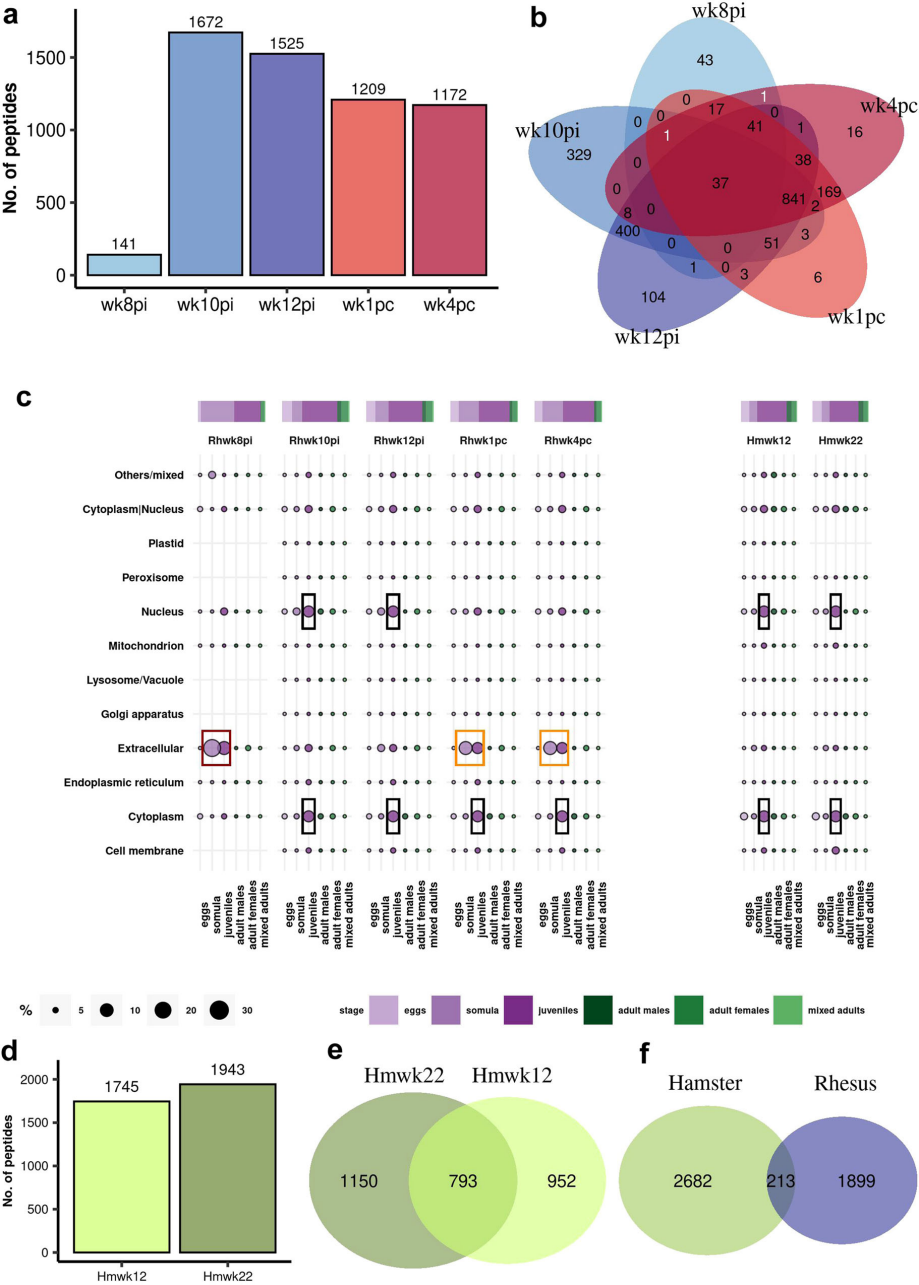
Enriched phage-displayed peptides captured by antibodies from rhesus macaques self-cured from *S. mansoni* infection and resistant to challenge compared with hamsters’ permissive control response. **a** Bar graph illustrating the number of enriched *S. mansoni* peptides determined by Bayesian statistical analysis of rhesus macaques’ PhIP-Seq data at each week post-infection (wk8pi to wk12pi) at the self-cure phase, and at weeks post-challenge (wk1pc, wk4pc), as described in Methods. Statistical significance (*P* ≥ 0.85). **b** Venn diagram demonstrating the overlap of enriched peptides in post-infection (wk8pi to wk12pi) and post-challenge (wk1pc, wk4pc) sequenced samples. Each color represents the set of peptides from a specific week, while the intersection in the center represents the 37 enriched peptides common to all five-time points. Two numbers in white indicate unique peptides overlapping wk8pi/wk1pc and wk8pi/wk4pc. **c** Proteins comprising the enriched peptides captured by rhesus macaques’ and hamsters’ antibodies were annotated using public data regarding *S. mansoni* life-stage expression and subcellular localization. The top bar and circle colors represent the life-cycle stages indicated in the legend at the bottom. The circle sizes indicate the percentage of enriched peptides in each subcellular location and life-cycle stage, as shown in the scale at the bottom. The colored boxes highlight important antibody targets from rhesus macaques and hamster immune response at different time points. **d** Bar graph illustrating the number of enriched *S. mansoni* peptides determined by Bayesian statistical analysis of PhIP-Seq data obtained from hamsters’ serum collected at wk12 and wk22 post-infection (Hmwk12 and Hmwk22). **e** Venn diagram demonstrating the overlap of enriched peptides in infected hamster samples at Hmwk12 and Hmwk22. **f** Comparison of peptides recognized by rhesus macaques and hamster antibodies, considering all sequenced time points in both species.

The first PhIP-Seq samples analyzed were from rhesus macaques’ plasma collected at week 8 post-infection (wk8pi), the earliest time point at which eggs and circulating anodic antigens (CAA) reached a peak and before parasites started dying^23^. Bayesian statistical analyses showed significant enrichment of 141 peptides (Fig. 3a). At week 10 post-infection (wk10pi), the time point when parasites started dying^23^, a marked increase to 1672 enriched peptides was observed, the highest number in the series (Fig. 3a). Samples from wk12pi, at the beginning of the phase of parasite clearance^23^, showed 1525 enriched peptides (Fig. 3a).

Rhesus macaques’ self-cure phase was followed up to wk42pi, at which point the macaques were challenged with 700 cercariae, the same number used at infection^23^. PhIP-Seq samples incubated with plasma from rhesus macaques collected at week one and week four post-challenge (wk1pc and wk4pc) showed 1209 and 1172 enriched peptides (Fig. 3a). Among these, 37 sequences from MEG-4.1 (as detailed below) were consistently detected across all sequenced time points, demonstrating their presence throughout the self-cure and resistance to challenge phases (Fig. 3b). Interestingly, only two distinct enriched peptides from wk8pi, namely from a cathepsin B (SmCatB) paralog (Smp_158420.1|307) and from an asparaginyl endopeptidase (SmAE) (Smp_075800.1|766) showed overlap exclusively with the post-challenge weeks (Fig. 3b, white numbers).

### Rhesus macaques’ antibodies from early phase infection recognized extracellular proteins highly expressed in early *S. mansoni* intra-mammalian life-stages

To gain insights into the subcellular location and life-cycle stage expression of proteins comprising the statistically enriched peptides, we annotated those proteins using available public data^29,30^, which revealed that rhesus macaque’s antibodies from wk8pi recognized the largest numbers of motifs from extracellular proteins expressed in the larval somula stage and juvenile worms (Fig. 3c, red box). Notably, at this specific time point, peptides from extracellular proteins accounted for 70% of all enriched targets (Supplementary Information, Supplementary Fig. 4).

A remarkable shift in the immune response pattern was observed in samples incubated with plasma from wk10pi and wk12pi (Fig. 3c, black boxes). This transition was characterized by a decrease in the targeting of extracellular proteins, now accounting for only 10 and 20% of all enriched peptides from these time points (Supplementary Information, Supplementary Fig. 4). Concurrently, there was a notable redirection of the IgG response towards intracellular proteins, such as nuclear and cytoplasmic proteins expressed throughout various intra-mammalian life stages, with a particular emphasis on the juvenile form (Fig. 3c, black boxes), indicating a loss in the parasite’s cellular integrity. Interestingly, samples from the challenge-resistant phase showed a boost response against peptides from extracellular proteins (Fig. 3c, orange boxes), representing approximately 40% of all detected peptides at both wk1pc and wk4pc (Supplementary Information, Supplementary Fig. 4).

Interestingly, hamsters exhibited a robust response against a high number of peptides at the two infection time points studied, with 1745 enriched peptides at wk12pi and 1943 at wk22pi (Fig. 3d) and a significant overlap of 783 targets among the two (Fig. 3e). Notably, the cellular compartments of proteins recognized by the hamsters’ sera collected at wk12pi and wk22pi, namely nuclear and cytoplasmatic compartments (Fig. 3c, right, black boxes), were intriguingly similar to the compartments recognized by rhesus macaques’ antibodies collected at wk10pi and wk12pi (Fig. 3c, left, black boxes). However, the number of overall peptides detected in common between rhesus macaques’ and hamsters’ samples was minimal, accounting for only 10.1% (213/2112) of the targeted peptides in the rhesus macaque model and 7.4% (213/2895) in the hamster model (Fig. 3f). This observation raises the possibility that the hamster’s response, a permissive host, may be targeting antigens associated with spurious parasite death rather than being coordinated as in the self-cure response mounted by non-permissive rhesus macaques. Involvement of the humoral response mounted against extracellular proteins in the parasite death will be further explored below in both infection models and, additionally, in an infected mice model.

### Comprehensive analysis of the detected *S. mansoni* enriched peptides from extracellular proteins

Here, we expanded our investigation to explore the diversity of extracellular proteins represented by the peptides captured by the total IgG antibody responses in the rhesus macaque model at the different assayed weeks. To accomplish this, we utilized available single-cell data^31^ to identify the organs where the genes encoding these various extracellular proteins are expressed.

**Fig. 4:**
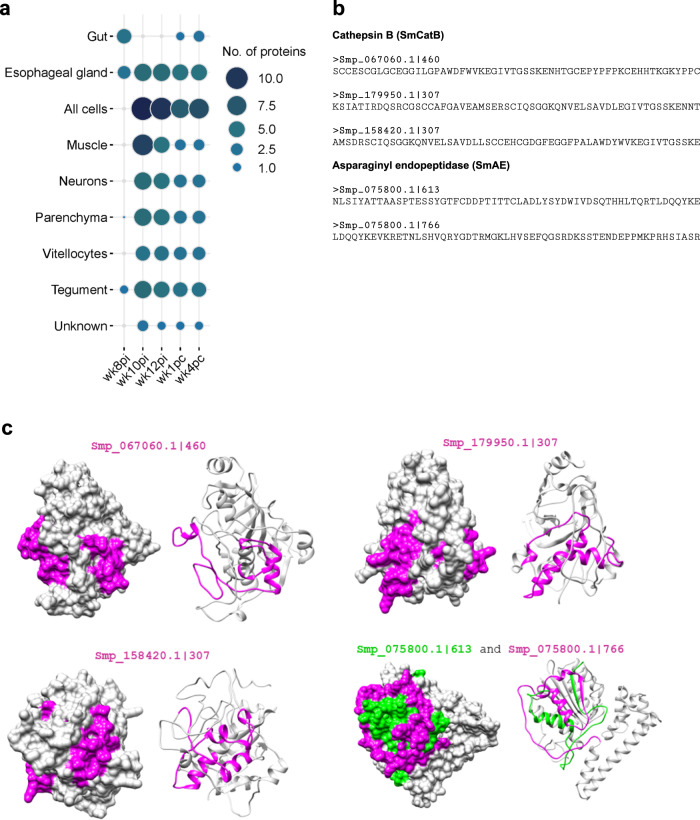
Expression localization of *S. mansoni* extracellular proteins containing the peptides captured by antibodies from self-cured and challenge-resistant rhesus macaques. **a** Bubble plot depicting the number of extracellular proteins comprising enriched peptides at predicted *S. mansoni* organ expression locations using publicly available single-cell data. Circle size and color range (legend on the right) represent the number of distinct proteins enriched in samples incubated with rhesus macaques’ plasma collected at each indicated week post-infection (wk8pi, wk10pi, wk12pi) or week post-challenge (wk1pc, wk4pc), or in samples incubated with hamsters’ sera collected at wk12pi or wk22pi, which are analyzed together here under the label “Hamster”. **b** The 58-mer enriched peptide sequences from three cathepsin B paralogs (Smp_067060.1|460, Smp_179950.1|3-7, Smp_158420.1|307) and two stretches from the asparaginyl endopeptidase (Smp_075800.1|613 and Smp_075800.1|766). These proteins are predicted as secreted in the parasite gut and begin to be recognized by the rhesus macaques’ plasma at wk8pi. **c** Homology models from predicted extracellular proteins expressed in the parasite gut highlighting enriched peptides on the specific protein surfaces. Protein structures are depicted in grey, while enriched peptides are color-coded in pink (representing most peptides) or green (specifically Smp_075800.1|613).

Our analysis unveiled a notable primary immune response from rhesus macaques’ plasma collected at wk8pi, specifically targeting four different extracellular proteins expressed in the gut and three in the esophageal gland of *S. mansoni* (Fig. 4a). These organs, readily accessible to the host immune system, play a crucial role in the parasite nutrient uptake^32,33^. The four gut proteins were three different SmCatB paralogs, each one represented by a different peptide (peptide IDs: Smp_067060.1|460, Smp_179950.1|307, and Smp_158420.1|307) (Fig. 4b), and one SmAE asparaginyl endopeptidase represented by two different peptides (peptide IDs: Smp_075800.1|613 and Smp_075800.1|766) (Fig. 4b), all of these peptides being predicted by homology modeling to reside at the exposed external surface of these proteins (Fig. 4c). Notably, SmAE peptide (ID: Smp_075800.1|766) is the single peptide from the gut detected at wk1pc (see Fig. 4a, and Supplementary Information, Supplementary Table 3) and also detected at wk4pc together with one SmCatB paralog (peptide ID: Smp_158420.1|307) (see Fig. 4a, and Supplementary Information, Supplementary Table 3).

Compared with wk8pi, plasma collected at wk10pi and wk12pi showed a broader response, targeting six different esophageal gland proteins and displaying a robust immune response against proteins expressed throughout all the body cells (Fig. 4a). Interestingly, among these proteins expressed in all cells, plasma collected from wk10pi, wk12pi, as well as wk1pc, and wk4pc consistently captured motifs from seven extracellular proteins, of which three are predicted as located at the external surface of cell membranes, namely Protein sax-3, probable peptidylglycine alpha-hydroxylating monooxygenase one and AP-3 complex subunit beta-2 transport protein (Supplementary Information, Supplementary Table 3). Other proteins are a putative disintegrin and metalloproteinase (ADAM) secreted enzyme, a granulin protein, and two hypothetical membrane proteins (Supplementary Information, Supplementary Table 3). Additionally, a recently annotated transmembrane cell adhesion receptor mua-3 protein expressed in the tegument is one of the four proteins consistently recognized in the tegument by rhesus macaque’s response from wk10pi onwards (Fig. 4a, and Supplementary Information, Supplementary Table 3).

Although the response mounted against proteins widely expressed throughout the body cells and in the tegument may impair the parasite’s survival within the rhesus macaque host, it is noteworthy that antibodies from wk10pi and wk12pi also recognized proteins predicted to be expressed in cells from internal organs, such as muscle, parenchyma, vitellocytes, and neurons (Fig. 4a, and Supplementary Information, Supplementary Table 3). This recognition suggests a potential disruption in the overall body structure of the parasite, thus releasing internal proteins, which aligns with our findings on subcellular localization. Of note, hamsters’ PhIP-Seq samples’ overall results (combined results from wk12pi and wk22pi) showed that a few peptides from extracellular proteins were recognized in common with rhesus macaques’ response (Supplementary Information, Supplementary Table 3). Namely, one protein expressed in the gut was detected as enriched, SmCatB (peptide ID: Smp_067060.1|460), along with five proteins from the esophageal gland (as further described below), one from the parenchyma, and two from tegument (Supplementary Information, Supplementary Table 3).

### Peptides from MEG proteins expressed in the esophageal gland of *S. mansoni* are enriched in the PhIP-Seq dataset

**Fig. 5:**
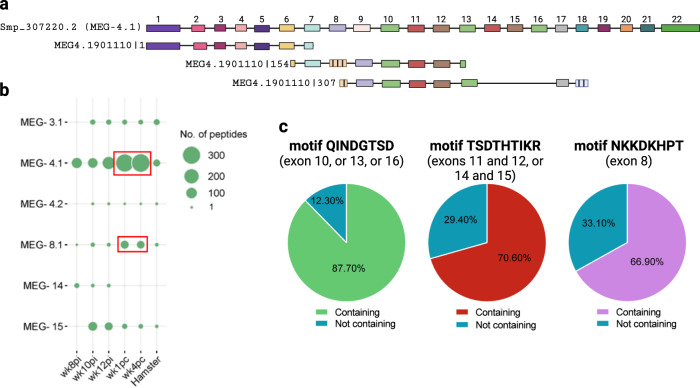
Enrichment of multiple MEG peptides originated from putative in silico designed alternatively spliced MEG isoforms. **a** Example of an in silico designed alternatively spliced variant of MEG-4.1. The upper sequence represents the canonical exons structure of Smp_307220.2 (MEG-4.1). Below, three in silico-designed 174-nt long oligo sequences are shown, which encode a putative splice variant, namely MEG4.1901110. The number following the pipe symbol (|) indicates the start position of the oligonucleotide segment encoding each peptide. Filled colored boxes represent exons in the canonical isoform, while hatched boxes represent non-canonical new exons. Exons are shown to scale, but intron lengths are not proportional for illustrative purposes. Numbers above exons indicate their position in the canonical sequence. Note the seven aa (21-nt) overlap between the first two consecutive oligo sequences (MEG4.1901110 | 1 and MEG4.1901110|154). The third, last sequence (MEG4.1901110|307) extends by a few amino acids the in silico predicted shorter MEG4.1 protein isoform, therefore having a longer overlap with the second oligo. **b** Number of enriched peptides per MEG family gene that were captured by rhesus macaques’ plasma samples collected at different weeks post-infection (wk8pi, wk10pi, wk12pi) or at weeks post-challenge (wk1pc, wk4pc), as well as the overall number of enriched peptides captured by infected hamster serum samples (Hamster). Circle size represents the number of peptides (legend on the right). Red boxes highlight the increase in MEG-4.1 and MEG-8.1 peptides captured by rhesus macaques’ plasma after challenge. **c** Pie charts illustrate the percentage of enriched peptides containing three different highly frequent motifs among all MEG-4.1 sequences captured with rhesus macaques’ plasma or not containing (legends at the bottom of each pie chart). Each motif sequence and the respective exon position within the Smp_307220.2 sequence is shown at the top of each pie chart.

Enriched peptides from six different proteins expressed exclusively at the parasite’s esophageal gland (Fig. 4a) belonged to MEG families; MEGs are a large *S. mansoni* family of proteins encoded by genes comprised of several symmetrical micro-exons (3 to 36 bp long) distributed in tandem^34^. We have included hundreds of different putative alternatively spliced MEG isoforms in the phage-display library, which were designed in silico, with the isoforms being annotated according to the family (Supplementary Information, Supplementary Table 4). For instance, for the MEG-4.1 family, the annotation of a phage-displayed peptide is MEG4.1901110|307, where the number following the name MEG4.1 is a code for a given isoform, and the number that follows the pipe symbol (|) indicates the start position of the oligonucleotide segment encoding that given peptide (Fig. 5a). Notably, we consistently detected many enriched peptides derived from MEGs across all tested weeks in rhesus macaques (Fig. 5b, and Supplementary Information, Supplementary Table 2). For each MEG gene, the set of enriched sequences consists of multiple peptides that share a similar or identical enriched motif, with splicing variations present in other regions of each different construct (see, for example, Fig. 5a, c).

At wk8pi, rhesus macaques’ plasma captured a few peptides from three distinct MEGs: MEG-4.1, MEG-8.1, and MEG-14 (Fig. 5b). Subsequently, starting from wk10pi onwards, the response expanded to include six different genes, namely MEG-3.1, MEG-4.1, MEG-4.2, MEG-8.1, MEG-14, and MEG-15, while excluding MEG-14 motifs at post-challenge weeks (Fig. 5b). Multiple alternatively spliced peptides were detected for each MEG gene in the sequenced samples. However, these peptides contained identical or highly similar reactive motifs (see, for example, Fig. 5c).

Interestingly, our analysis of enriched MEG-4.1 peptides across rhesus macaques’ sequenced samples revealed the presence of frequent canonical motifs (Fig. 5c). Notably, motif QINDGTSD was found in 87.70% (286/326) of the captured sequences. This motif is encoded within three copies at exons 10, 13, and 16 from Smp_307220.2 isoform (Fig. 5c).

Motif TSDTHTIKR was also notable, present in 70.60% (230/326) of the sequences (Fig. 5c). It is encoded within two copies at the exon junctions between exons 11 and 12 and between exons 14 and 15. Additionally, motif NKKDKHPT was identified in 66.90% (218/326) of the captured sequences and is encoded at exon 8 (Fig. 5c).

Therefore, variation in the number of captured peptides from a given MEG gene across different weeks (Fig. 5b) suggests a larger population of specific antibodies targeting multiple copies of the same antigen encoded in the phage-display library. A stronger response was observed at post-challenge weeks (wk1pc and wk4pc) when more peptides from MEG-4.1 and MEG-8.1 genes were captured compared to earlier time points (Fig. 5b, red boxes).

Notably, hamsters exhibited a comparatively lower response to these MEG genes, with only a limited number of sequences enriched from MEG-3.1, MEG-4.1, MEG-4.2, MEG-8.1, and MEG-15 (Fig. 5b). Comparison between MEG-4.1 reactive motifs between rhesus macaques and hamsters showed no overlap, as shown further below.

### Validation of PhIP-Seq results with peptide microarrays

We designed and ordered a peptide microarray to map the reactive motifs within the PhIP-Seq enriched 58-aa peptides, which were recognized by antibodies against *S. mansoni* gut and esophageal gland proteins during self-cure and challenge-resistant phases. Each of the gut protein peptides (SmCatB Smp_158420.1|307, SmCatB Smp_179950.1|307, SmAE Smp_075800.1|613, and SmAE Smp_075800.1|766) and esophageal gland proteins (MEG-4.1, MEG-8.2, MEG-3.1, and MEG-15) were represented in the peptide microarray by a set of 15-mer peptides that covered with 2-amino acid offset the entire set of 58-amino acid sequences.

**Fig. 6:**
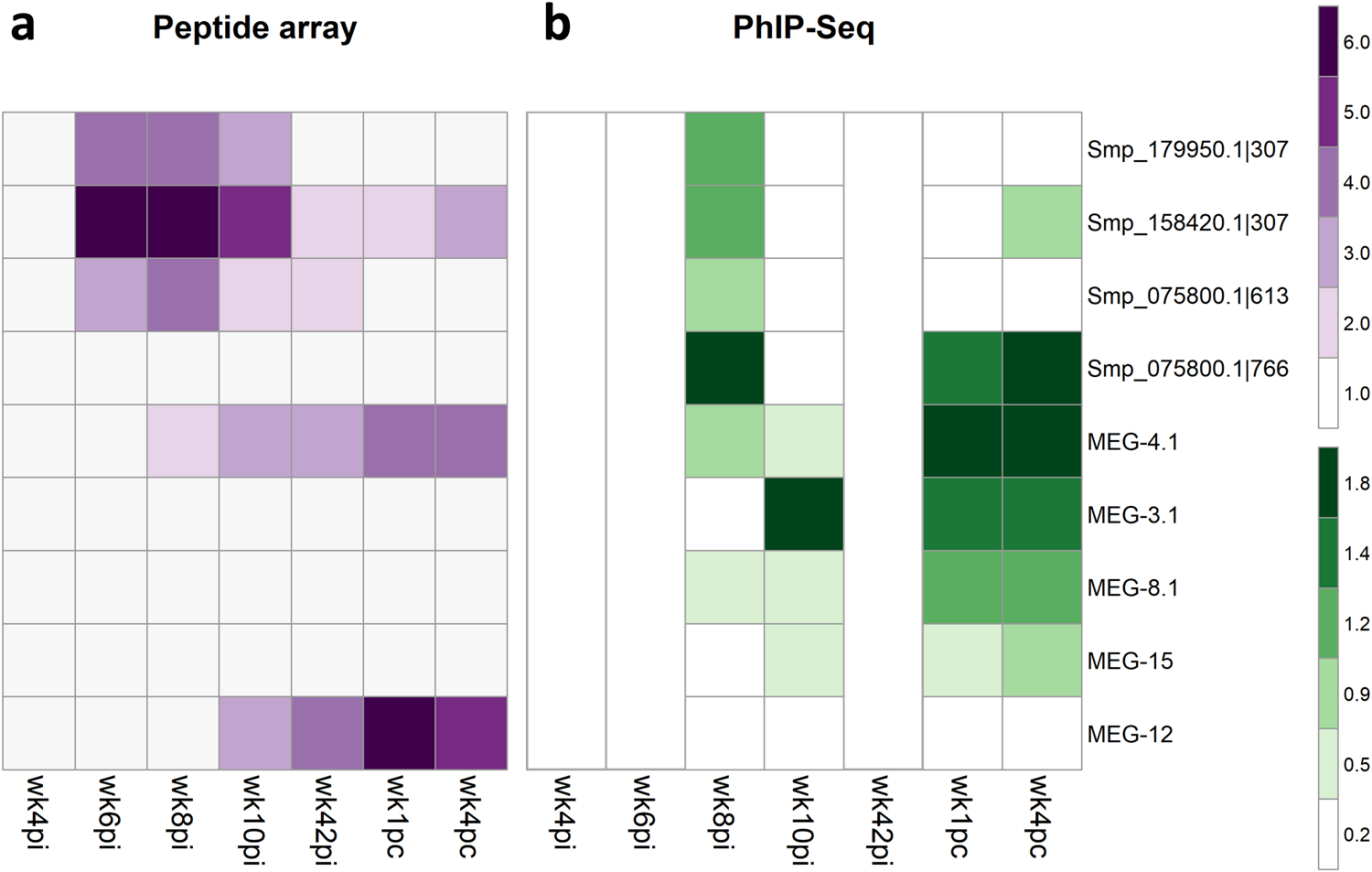
Heatmap of significantly reactive 15-mer peptides recognized by antibodies in the plasma from rhesus macaques self-cured and challenge-resistant to *S. mansoni*. Each line represents one peptide from a different protein or multiple peptides from the indicated MEG family member’s alternatively spliced MEG protein variants. These were selected for the peptide microarray based on their enriched signal in the PhIP-Seq analysis. **a** Median intensity signal in the peptide microarray among the different rhesus macaques and across the entire set of 15-mer peptides is shown in purple (see color scale) for those entries with significantly reactive peptide motifs, as determined with the pepStat tool. Columns represent the four time points at weeks post-infection (wk4pi, wk6pi, wk8pi, wk10pi), the time point at challenge (wk42pi), and the two time points at weeks post-challenge (wk1pc, and wk4pc). In addition, MEG-12, not enriched in the PhIP-Seq analysis, was included in the peptide microarray. **b** For comparison, the median enrichment values among the different rhesus macaques of significantly enriched peptides in the PhIP-Seq assay are shown in green (see color scale). Empty columns represent time points not assayed by PhIP-Seq.

The peptide microarray was screened with rhesus macaques’ plasma samples from wk4pi, wk6pi, wk8pi, and wk10pi, as well as from wk42pi (challenge week), and wk1pc and wk4pc (Fig. 6a). The statistically significant signal intensities above the baseline response at wk0 (pre-infection) were computed using the pepStat analysis pipeline. The IgG median response intensities across all rhesus macaques each week are shown (Fig. 6a). We also compared the median intensity of the response obtained in the PhIP-Seq assay (wk8pi, wk10pi, wk1pc, and wk4 pc) (Fig. 6b).

During the pre-patent period at wk4pi, migrating somula did not elicit a significant early IgG response against any of the selected peptides from gut and esophageal gland proteins (Fig. 6a). Of note, by wk6pi, when the expression of gut and esophageal gland proteins in juveniles had increased, the antibodies in rhesus macaques’ plasma recognized stretches from both SmCatB peptides (Smp_179950.1|307, and Smp_158420.1 | 307) and from SmAE asparaginyl endopeptidase (Smp_075800.1|613) screened on the array (Fig. 6a). In addition to these targets, plasma from wk8pi recognized MEG-4.1 epitopes (Fig. 6a), validating the PhIP-Seq analysis for these antigens at this specific time point (Fig. 6b). On the other hand, wk8pi plasma failed to recognize SmAE peptide Smp_075800.1|766 (Fig. 6a). However, its reactivity was confirmed through both PhIP-Seq (Fig. 6b) and Dot Blot assays (Supplementary Information, Supplementary Fig. 5).

By wk10pi, at the rhesus macaques-initiated parasite clearance phase^23^, sustained antibody recognition of linear epitopes from SmCatBs (Smp_179950.1|307, and Smp_158420.1|307) and SmAE (Smp_075800.1|613) was observed (Fig. 6a). However, the PhIP-Seq data from this time point did not reveal a response against gut proteins (Fig. 6 b), pointing to a possible lack in sequencing depth after the exposure and capture of multiple antigens at this time point. Of note, only MEG-4.1 remained detected on the peptide microarray at wk10pi (Fig. 6a), showing concordance with PhIP-Seq (Fig. 6b). In contrast, the other MEGs detected by PhIP-seq at wk10pi were not reactive on the peptide microarray.

Notably, just before the challenge at wk42pi, a significant response against SmCatB Smp_158420.1|307, SmAE Smp_075800.1|613, and MEG-4.1 epitopes was observed (Fig. 6a). Epitopes from SmCatB Smp_158420.1|307 and MEG-4.1 remained significantly recognized at wk1pc and wk4pc (Fig. 6a), suggesting their potential role in blocking reinfection.

Rhesus macaques’ plasma from all assayed weeks were not reactive against MEG-3.1, MEG-8.1, and MEG-15 on the peptide microarray (Fig. 6a), being only identified using the PhIP-Seq method (Fig. 6b). It is worth noting that in the PhIP-Seq assay, a 58-mer peptide was displayed, and it is possible that these phage-displayed peptides assumed a conformational structure that was not captured by the linear peptides printed on the peptide microarray.

Interestingly, MEG-12 (MEG000012|1) had not been enriched in the PhIP-Seq assay, showing low read counts (Supplementary Information, Supplementary Table 1). Therefore, we included it in the peptide microarray as a putative negative control. Curiously, macaques’ plasma from wk10pi onwards strongly recognized MEG-12 (Fig. 6a). This unexpected finding suggests a potential false negative result in the PhIP-Seq analysis, possibly due to the low abundance in the input library of phages displaying this antigen and/or an unfavorable folding of the peptide in the fusion protein displayed by the phage.

These observations underscore the successful validation of the rhesus macaque’s antibody response against gut proteins and MEG-4.1, both during the self-cure and the challenge-resistant phases.

### SmCatB and SmAE epitope mapping of rhesus macaques’ plasma reactivity and comparison with non-permissive hosts

**Fig. 7:**
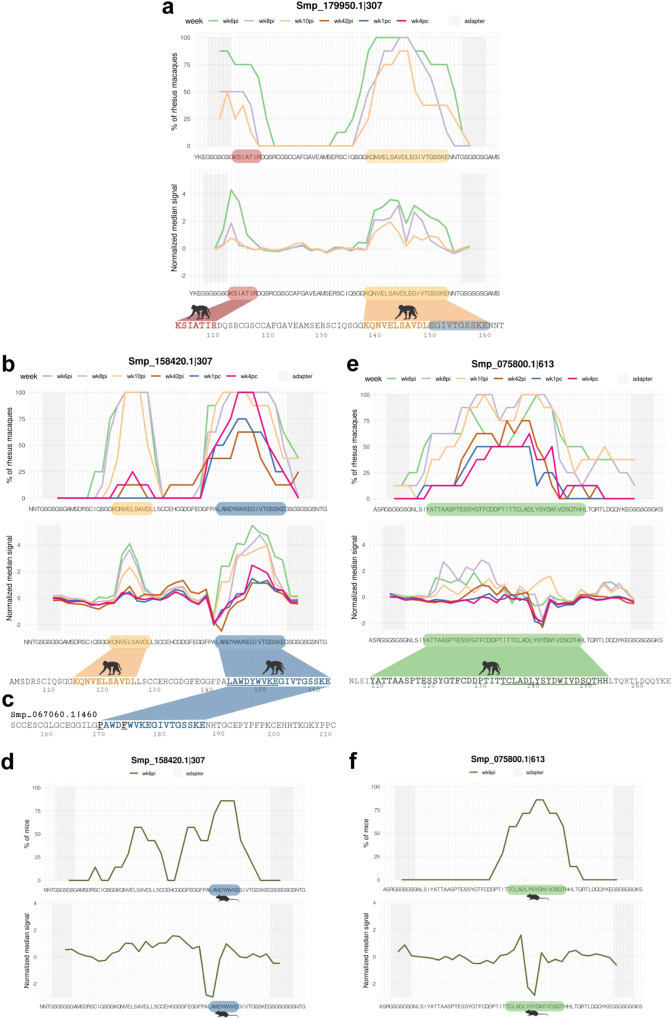
*S. mansoni* SmCatB paralogs (Smp_179950.1 and Smp_158420.1) and SmAE Asparaginyl endopeptidase (Smp_075800.1) epitope mapping with reactive plasma from rhesus macaques. **a**, **b**, **and e** For each 58-mer phage-peptide indicated at the top of each panel, its peptide sequence is shown at the bottom of each chart; microarray adapter stretches at both ends of the sequence are highlighted with a grey background. The upper chart represents the percentage of animals (out of 8 rhesus macaques) exhibiting antibody reactivity against epitopes from that peptide, and each line color represents a different week post-infection (wk6pi, wk8pi, wk10pi, wk42pi) or post-challenge (wk1pc and wk4pc) (legends on top of upper charts). The lower chart represents the median normalized signal intensities at each week post-infection/challenge. The colored blocks at the lowermost sequence in each panel highlight the epitopes recognized by at least 50% of rhesus macaques (out of 8 animals); part of the motif that is underlined is in common with the motif recognized by sera from infected mice (see below). **c** Another PhIP-Seq enriched SmCatB paralog, namely peptide Smp_067060.1|460, which was not screened in the peptide array, is shown here for comparison with Smp_158420.1|307 sequence. Common epitopes between the two different SmCatB sequences are marked in blue, and the two different amino acid residues between both motifs are shown in (**c**) in black font and underlined. **d**, **f** Upper charts showing the fraction of mice (out of 7 animals) exhibiting week 6 post-infection (wk6pi) serum reactivity against an epitope from that peptide sequence. Lower charts represent the median normalized signal intensities. Images created with Biorender.com.

Antibodies from all rhesus macaques strongly recognized epitopes from SmCatB paralogs (Smp_179950.1|307 and Smp_158420.1|307) at wk6pi (Fig. 7a, b). Smp_179950.1|307 was recognized at two distinct motifs: the 7-aa residues KSIATIR close to the N-terminal (wk6pi and wk8pi) (Fig. 7a, red color), and the 21-aa sequence KQNVELSAVDLEGIVTGSSKE close to the C-terminal (wk6pi, wk8pi, and wk10pi) (Fig. 7a, orange color). Among the SmCatB Smp_158420.1|307 epitopes, residues LAWDYWVKEGIVTGSSKE (18-aa) (Fig. 7b, blue color) showed the highest median signal reactivity during the initial self-cure weeks (Fig. 7 b, green, purple and orange curves), followed by a recall response at wk4pc (Fig. 7b, pink curve). Epitopes KQNVELSAVDL (11-aa) (Fig. 7a, b, orange) and EGIVTGSSKE (10-aa) (Fig. 7a. b, blue) were recognized in both paralogs, Smp_179950.1|307 and Smp_158420.1|307, but at different positions within these sequences (Fig. 7a, b). The EGIVTGSSKE motif is contained within the longer 18-aa epitope (Fig. 7b, blue color motif).

The PhIP-Seq enriched peptide SmCatB Smp_067060.1|460 was not included in the peptide microarray, and it is interesting to note that it shares a very similar sequence with the 18-aa epitope of SmCatB Smp_158420.1|307, only differing by two amino acids (P and F) as marked in Fig. 7c.

We compared the antibody response of rhesus macaques with the response from two different permissive controls, infected hamsters, and mice. Interestingly, hamsters did not show IgG response against cathepsins (not shown). In contrast, sera from infected mice recognized a short 9-aa motif LAWDYWVKE (Fig. 7d, see blue-colored sequence), which is part of one of the motifs recognized by plasma from rhesus macaques (Fig. 7b, see the blue-underlined sequence), with the mouse response being triggered in 57.1% (4/7) to 85.7% (6/7) of the animals (Fig. 7d).

The PhIP-Seq enriched peptide Smp_075800.1|613 from SmAE asparaginyl endopeptidase protein, when tested in the peptide microarray, was recognized almost throughout all its sequence by plasma from at least 50% (4/8) of rhesus macaques, covering 42 amino acid residues (Fig. 7e, green sequence). A prominent 35-aa sequence was recognized by at least 62.5% (5/8) of the macaques, with the highest median signal observed at wk8pi (Fig. 7e, purple curve), followed by wk6pi and wk10pi, resulting in a response from all rhesus macaques against some of the residues. A lower signal was observed in 50% (4/8) of the macaques at wk1pc and wk4pc (Fig. 7e, blue and pink curves). When infected mice, the permissive host, was tested, a shorter reactive motif TCLADLYSYDWIVDSQT (17-aa) was detected (Fig. 7f, green sequence), with a response in 57.1 (4/7) to 85.7% (6/7) of the animals. This motif is part of the longer motif recognized by rhesus macaques’ plasma (Fig. 7e, see the green-underlined sequence).

### Species-specific motifs identified in MEG-4.1 by epitope mapping

A robust immune response against MEG-4.1 peptides encoded by new exons of MEG-4.1 was detected with the peptide microarray, which was employed to investigate the antibody response of self-cured and challenge-resistant rhesus macaques and to compare it with the antigenic determinants recognized by infected permissive hosts. These new exons on MEG-4.1 Smp_163630 v5.2 gene, the version that was used for the phage-display library, were annotated using our in silico predicted alternatively spliced MEG library sequences and were confirmed with MEG-4.1 Smp_307220 sequence, on the updated *S. mansoni* transcriptome version 7. As the rhesus macaques’ antibodies captured over 300 MEG-4.1 58-mer PhIP-Seq enriched peptides, the peptide microarray was designed to cover the 52^nd^ to the 149^th^ amino acid positions from Smp_307220.2, plus non-canonical exon junctions, contemplating possible epitopes represented in the PhIP-Seq enriched peptides (Supplementary Information, Supplementary Table 6).

**Fig. 8:**
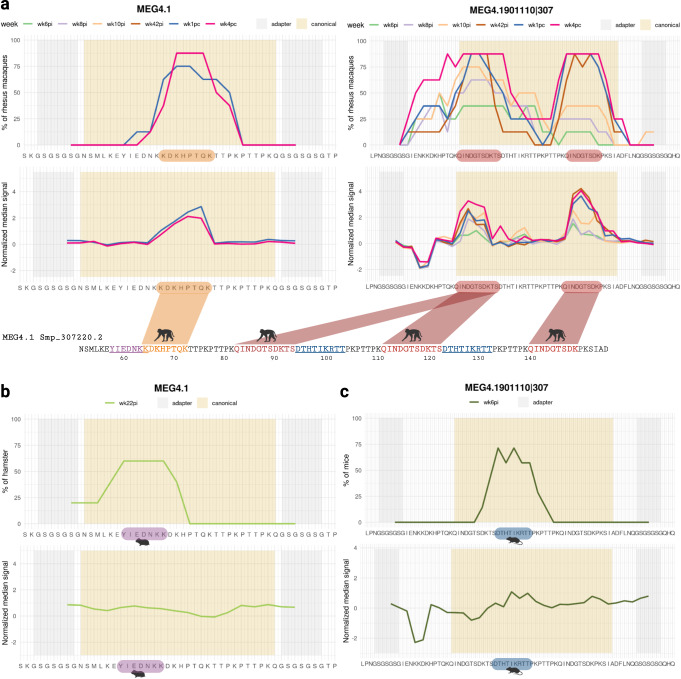
*S. mansoni* MEG-4.1 epitope mapping with reactive plasma from rhesus macaques. **a** A peptide segment from MEG-4.1 (Smp_307220.2) (left panels) and a 58-mer phage-peptide from a MEG-4.1 in silico-designed splice variant (right panels) that were included in the peptide microarray are shown at the bottom of each chart; microarray adapter stretches at both ends of the sequences are highlighted with a grey background, while canonical positions have a salmon background. The upper charts represent the percentage of rhesus macaques (out of 8 animals) exhibiting antibody reactivity against epitopes from that peptide, and each line color represents a different week post-infection (wk6pi, wk8pi, wk10pi, wk42pi) or post-challenge (wk1pc and wk4pc) (legends on top of upper charts). The lower charts depict the median normalized signal intensities at each week post-infection/challenge. The colored blocks at the lowermost sequence in each panel highlight the epitopes recognized by at least 50% of rhesus macaques (out of 8 animals); some motifs that are underlined were recognized by sera from infected hamsters or infected mice, and each color denotes distinct epitopes or different species (see below). **b** Upper chart shows the fraction of hamsters (out of 5 animals) exhibiting week 22 post-infection (wk22pi) serum reactivity against an epitope from that peptide sequence. Lower chart represents the median normalized signal intensities. **c** Upper chart shows the fraction of mice (out of 7 animals) exhibiting week 6 post-infection (wk6pi) serum reactivity against an epitope from that peptide sequence. The lower chart represents the median normalized signal intensities. Images created with Biorender.com.

Rhesus macaques’ antibodies significantly recognized two canonical MEG-4.1 epitopes present in six sequences designed in the peptide microarray (Fig. 8a, and Supplementary Information, Supplementary Fig. 6). A critical response of 75% of the rhesus macaques against epitope KDKHPTQ was observed with plasma from post-challenge at wk1pc (Fig. 8a, left panels); the median signal response slightly decreased at wk4pc, and a higher number of animals (87.5%) had antibodies against this motif (Fig. 8a, left panels). Looking at other segments of MEG-4.1 represented in the MEG4.1901110|307 peptide, a broad response against a different epitope was detected at all time points from wk8pi onwards, namely epitope QINDGTSDK (9-aa) (Fig. 8a, right panels), which was duplicated in this peptide sequence. From the challenge (wk42pi) until wk4pc, slightly different segments of this epitope were recognized by 87.5% (7/8) to 100% (8/8) of rhesus macaques, with a median signal intensity higher than that of earlier weeks (Fig. 8a, right panels, and Supplementary Information, Supplementary Fig. 6). Noteworthy, from all 333 PhIP-Seq enriched MEG-4.1 peptide sequences in the rhesus macaques’ dataset, only seven peptides (2.1%) do not contain any of these two highly reactive epitopes.

Infected hamsters and mice, permissive hosts, were tested and we found that 60% of hamsters recognized the YIEDNKK sequence (Fig. 8b). In comparison, ≥57% of mice responded to the canonical epitope’s residues DTHTIKRTT (Fig. 8c). Most interestingly, both motifs differ from those recognized by rhesus macaques (Fig. 8a), as indicated by the animal silhouettes at the bottom of Fig. 8a–c. Of note, a response against non-annotated putative alternatively spliced exon junctions was not significantly detected.

### MEG-12 epitopes mapping

**Fig. 9:**
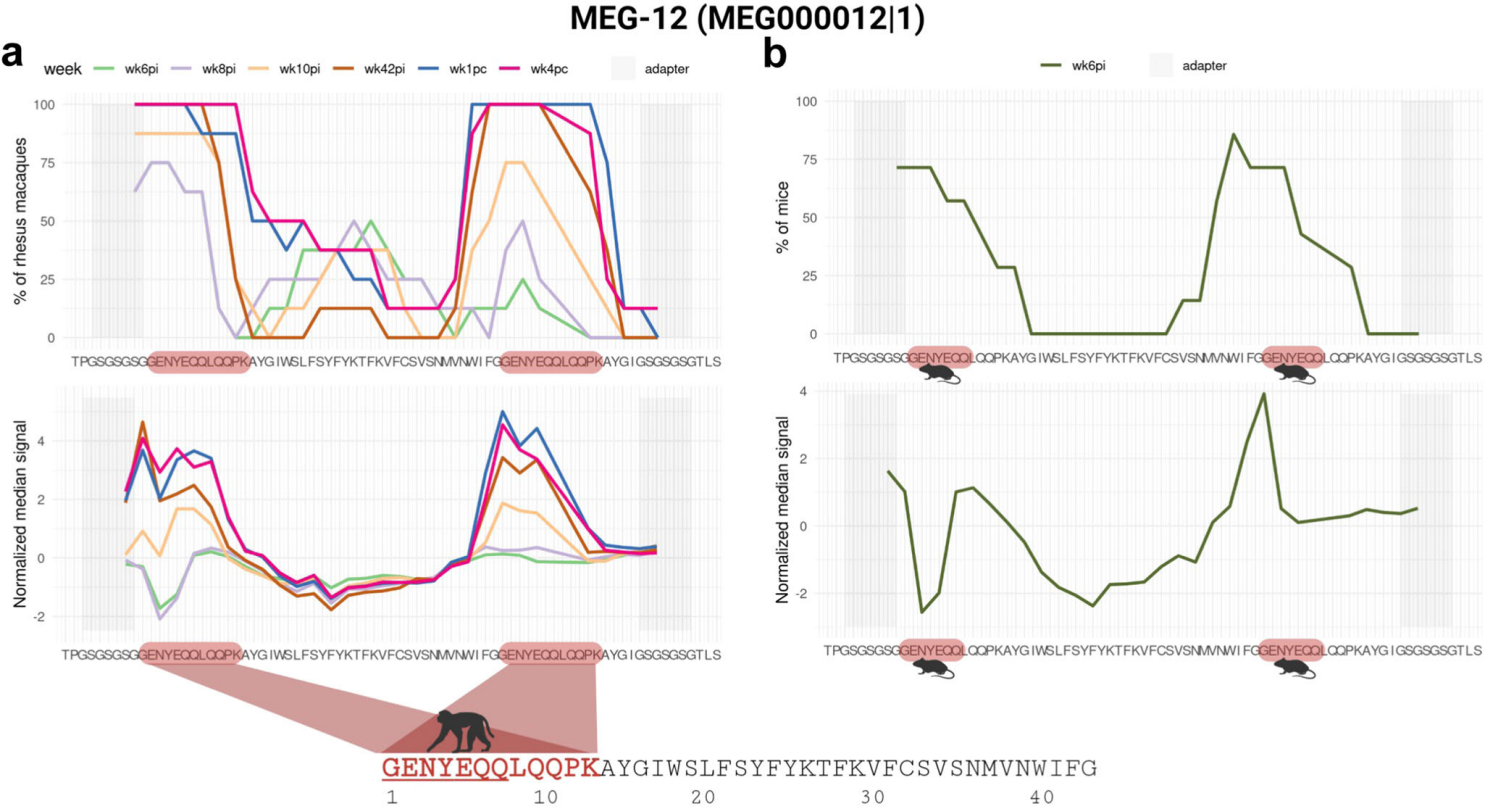
*S. mansoni* MEG-12 epitope mapping with reactive plasma from rhesus macaques. **a** The MEG-12 58-mer phage-peptide sequence is shown at the bottom of each chart; microarray adapter stretches at both ends are highlighted with a grey background. Note that the 16-aa N-terminal sequence of MEG-12 is duplicated at the end of the 42-aa original sequence in this phage-display peptide construct. The upper chart represents the percentage of rhesus macaques (out of 8 animals) exhibiting antibody reactivity against an epitope from that peptide, and each line color represents a different week post-infection (wk6pi, wk8pi, wk10pi, wk42pi) or post-challenge (wk1pc and wk4pc) (legends on top of upper charts). The lower chart represents the median normalized signal intensities at each week post-infection/challenge. The colored block at the lowermost sequence highlights the epitope recognized by at least 50% of rhesus macaques (out of 8 animals); part of the underlined motif is in common with the motif recognized by sera from infected mice (see below). **b** Upper chart shows the fraction of mice (out of 7 animals) exhibiting week 6 post-infection (wk6pi) serum reactivity against an epitope from the MEG-12 peptide sequence. The lower chart represents the median normalized signal intensities. Images created with Biorender.com.

MEG-12 is a pre-protein with 66-aa (Smp_152630), and the mature secreted sequence is only 42-aa long (Fig. 9a, bottom). The phage-display library contained 58-mer peptide inserts; consequently, the 42-aa long original MEG-12 sequence was designed on the library with an extended C-terminal sequence that included a duplicated N-terminal 16-aa sequence to make up for the 58-mer. The peptide microarray followed the same design and included the N-terminal duplicated sequence. Analysis of reactive epitopes within the MEG-12 protein on the peptide microarray revealed a strong median signal intensity of rhesus macaques’ antibodies against an N-terminal 12-aa motif GENYEQQLQQPK with rhesus macaques’ plasma from wk10pi onwards (Fig. 9a), showing slightly higher median intensities at wk1pc and wk4pc. Interestingly, a shorter epitope GENYEQQ was recognized by sera from mice in approximately 71.4% (5/7) of infected animals (Fig. 9b).

### Immunization of mice with peptides from *S. mansoni* blood-feeding and nutrient uptake proteins reduces female worm burden in a protection assay

**Fig. 10:**
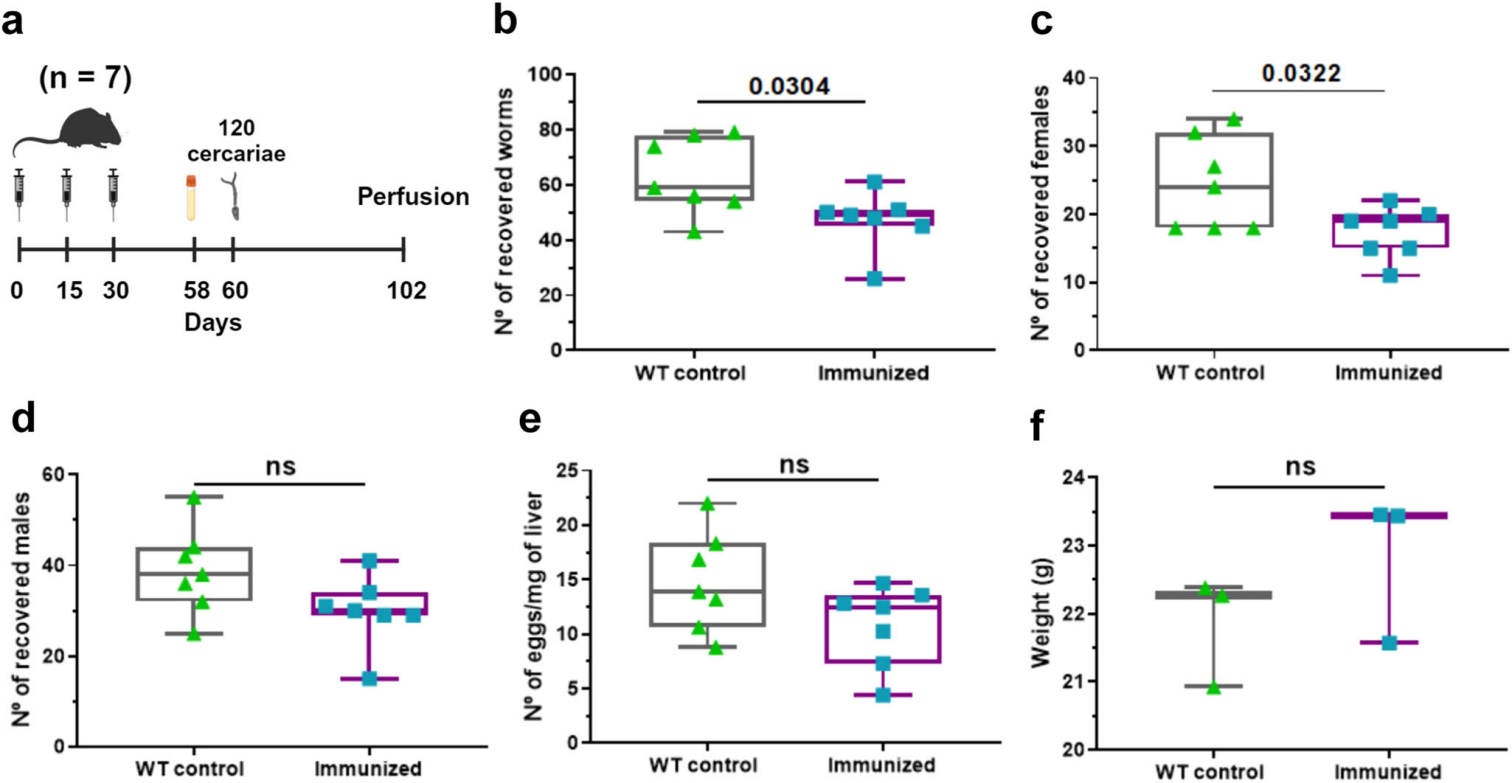
Protection assay with a pool of *S. mansoni* peptides from blood-feeding and nutrient uptake proteins. **a** Balb/c mice (*n* = 7 in each group) received three doses containing WT phage (no insert controls) or a pool of phages expressing SmCatB, SmAE, MEG-3.1, MEG-4.1, MEG-8.1, and MEG-15 peptides. Twenty-eight days after the third dose, serum was collected. The animals were challenged with 120 cercariae of *S. mansoni* each, on the 60th day, and perfused 42 days after infection. The following parameters were evaluated: **b** total number of worms recovered per mouse in the perfusion, **c** number of female parasites recovered per mouse, **d** number of male parasites recovered per mouse, **e** number of eggs recovered per milligram of mouse liver, and **f** weight of mouse in grams at the day of perfusion. Boxplot represents median, first and third quartiles, whiskers extend up to minimum and maximum. These parameters were compared between immunized animals and WT controls using unpaired t-test (*p* < 0.05). Images created with Biorender.com.

Based on the PhIP-Seq analysis, a set of peptides was selected as potential vaccine candidates, expressed individually in phages, and evaluated as a mixed pool in mice. Specifically, our selected pool included peptides from SmAE asparaginyl endopeptidase protein, SmCatB paralogs, MEG-3.1, MEG-4.1, MEG-8.1, and MEG-15. Seven mice were immunized with three doses of the pool of phage-displayed peptides that were intra-peritoneally inoculated at intervals of 15 days between doses. Immunized animals were infected with 120 cercariae per animal 30 days after the last dose and were perfused 42 days after infection (Fig. 10a). The results indicated that mice immunized with *S. mansoni* peptides had a significantly lower burden of adult worms than the wild-type (WT) control group (Fig. 10b). The worm burden reduction was 22% in the immunized group (Fig. 10b), with a marked decrease in the number of retrieved female worms (Fig. 10c). A non-significant reduction in male worms (Fig. 10d) and in the number of eggs in the liver (Fig. 10e) were seen. Additionally, there was no significant change in weight between the control and immunized mice groups (Fig. 10f).

Most interestingly, the peptide microarray screening with sera collected on the 58th day from 4 of the 7 animals revealed that the vaccination protocol did generate specific antibodies against two of the selected antigens (Supplementary Information, Supplementary Fig. 7), namely epitope FPALAWDYV from SmCatB Smp_158420|307 and LADLYSYDWIVDSQT from SmAE asparaginyl endopeptidase Smp_075800.1|613, which are epitopes similar to those recognized by self-cured rhesus macaques and infected mice; these were recognized by serum from ≥50% of vaccinated mice (Supplementary Information, Supplementary Fig. 7).

## Discussion

In the present study, we tracked the unique rhesus macaques IgG response against *S. mansoni* during the self-cure and challenge-resistant phases using a large synthetic DNA phage-display library that permitted an unbiased screening of an impressive 99.6% of all proteins known to be expressed across all parasite life-cycle stages. Previous works have successfully identified several potentially protective and safe schistosomiasis vaccine antigens by screening of sera from naturally resistant human individuals, using a protein array comprised of 217 selected, mostly surface-derived *S. mansoni* proteins^35^; in addition, two mimic antigenic epitopes have been identified by screening of sera from rats resistant to *S. japonicum* infection, using a phage-display 12-mer random peptide library^36^. The novel PhIP-Seq technique^25–27^ used here represents a significant improvement over previous approaches, which were limited to covering a small fraction of antigens rationally selected using recombinant protein expression^6,35,37–39^ or peptide microarrays^8,40^. Phage-displayed *S. mansoni* antigens screening employed here aligns with successful applications of phage-display libraries in identifying crucial vaccine candidates for diverse pathogens, including *Trypanosoma cruzi*^41,42^, SARS-CoV-2^43^, and *Leptospira* spp^44^.

Our findings reveal an earlier timeline for rhesus macaques’ immune defenses against *S. mansoni* than previously reported^22–24^, accompanied by significant changes in the antibody response along the self-cure phase. Thus, as early as the beginning of egg deposition (wk6pi)^23^, we observed a notable increase in antibodies targeting SmCatB, SmAE, and MEGs, essential proteins involved in parasite nutrient uptake^45,46^. Subsequently, from wk10pi onwards, there was a distinct shift in the antibody response towards intracellular antigens, probably coinciding with the loss of parasite integrity within its cells and tissues. This transition suggested a progression towards parasite clearance as antigens became exposed beyond the host-parasite interface^32^. Indeed, the lack of hemozoin pigment in the gut of damaged parasites recovered from self-cured rhesus macaques highlights the disruption of digestive functions along the entire *Schistosoma* digestive tract within this non-permissive host^23,24^.

In this study, we observed a significant immune response simultaneously developing at wk6pi against similar motifs among SmCatB paralogs, which are crucial proteins for hemoglobin digestion, and against SmAE, a proteolytic asparaginyl endopeptidase enzyme involved in activating cathepsins^46^. The rhesus macaques’ immune response strongly correlated with the juvenile blood-feeding stage (wk6pi), indicating the importance of simultaneously targeting these proteins during this critical early phase of parasite development.

Besides gut proteins, a robust immune response targeting the parasite esophageal gland was observed, specifically directed towards MEG proteins. In rhesus macaques, *S. mansoni* MEG proteins elicit a robust IgG immune response, which is believed to be a primary mechanism hindering parasite survival^22,24,32,40^. Notably, MEG-4 has been shown to be crucial in allowing the parasite to evade the susceptible host immune system successfully^47^. Here, rhesus macaques’ antibodies were shown to consistently recognize MEG epitopes throughout self-cure and challenge-resistant phases, demonstrating their immunogenicity. Interestingly, the discordant species-specific recognition of MEG-4.1 epitopes in rhesus macaques, mice, and hamsters identified here suggests potential variations in the host immune systems or possible differences in genetic backgrounds between permissive and non-permissive hosts^48^.

Remarkably, MEG-12, initially classified as a PhIP-Seq negative sequence, displayed a reactive epitope recognized by both rhesus macaques’ plasma and mice sera in the peptide microarrays. The epitope is identical to that recognized by infected children from an endemic area in Ethiopia^49^, and by mice immunized with attenuated cercariae^8^. This finding highlights MEG-12 as a well-established antigenic target^49^ and underscores a limitation of the PhIP-Seq technique in detecting low-copy antigens in the phage-display library.

Encouragingly, immunization with selected antigens expressed in phage has generated antibodies against SmCatB and SmAE in mice, causing a significant reduction in total worm burden by 22% and in females worm burden by 25%. Reduction in worm burden aligns with previous research with *S. mansoni*, where immunization of mice with full length rSmCatB and rSmCatL alone or combined have elicited a 54-65% worm burden reduction^50–52^, immunization with multi-antigenic-peptide constructs comprising two epitopes from CatB and CatL elicited significant 39% reduction in challenge worm burden with an opposite effect of 64% increase in liver eggs^53^, while immunization with full length rSmCatB led to an impressive >80% reduction in worm burden in mice^54,55^; of note, immunization of mice with rSmAE resulted in a 37% decrease in egg-laying^56^. Generating an effective vaccine against *S. mansoni* has been challenging. Nevertheless, introducing new schistosomiasis vaccines will become an essential component for disease elimination^57^. Eventually, more encouraging results could be reached with *S. haematobium*, for which an immunization of mice with CatB plus CatL adjuvanted with GAPDH caused a highly significant reduction of 72% in challenge worm burden and no eggs present in liver^58^, thus exhibiting a more successful protection than with *S. mansoni*, that showed 69% reduction in worm burden and only a reduction of 73% in liver eggs^52^.

Although low titers of specific antibodies were detected in phage-immunized mice in the present pilot vaccine trial, the phage-based vaccine has been proven a good antigen delivery system for *S. japonicum*^28^ and, more recently, for SARS-CoV-2^59^. We suggest that optimization of multi-antigen formulations, including SmCatB and SmAE peptide epitopes, other delivery systems, and adjuvants, is warranted. Additionally, MEGs are known to possess intrinsically disordered domains^60^ and should be further investigated, given that intrinsically disordered proteins with tandem repeat sequences have been shown to induce neutralizing antibodies against *Plasmodium falciparum*^61^.

In conclusion, our unbiased approach significantly contributed to a more holistic perspective on the intricate relationship between the parasite and the non-permissive rhesus macaque host immune response during the self-cure and resistance to challenge processes. By employing multiple immunomics techniques, these findings strongly support the potential of a combination of epitopes from a selected set of secreted-feeding proteins as highly promising vaccine candidates for schistosomiasis.

## Methods

### Ethics statement

Housing conditions of the rhesus macaques and experimental protocols used in the study were in strict accordance with the Ethical Principles in Animal Research adopted by the Conselho Nacional de Controle de Experimentação Animal (CONCEA) and were approved by the Institutional Animal Care and Use Committee of Instituto Butantan (CEUAIB 1388/ 15). The study was carried out in compliance with the ARRIVE guidelines. The design and execution of the study complied with the recommendations of the Weatherall report (2006) “The use of non-human primates in research”, in which there are sections dealing with the continued need for primates in schistosomiasis research, particularly in vaccine development. The study also complied with principles set out in the UK NC3Rs Guidelines Primate accommodation, care and use (revised version, October 2017) (http://www.nc3rs.org.uk/primatesguidelines). Twelve adult female rhesus macaques (*Macaca mulatta*) from the captive-breeding colony at the Central Animal Facility at Butantan Institute were group-housed for the whole experiment, permitting social interactions, continuous socialization and colony welfare, as detailed in our previous study^23^. Animal ages ranged from 7 to 16 years-old and the animals underwent hematological, parasitological, and clinical tests before the beginning of experiments^23^. The facility is accredited by Conselho Nacional de Controle de Experimentação Animal (CONCEA).

For permissive hosts, housing conditions of the Syrian hamsters (*Mesocricetus auratus*) and experimental procedures used in this study were also in strict accordance with the Ethical Principles in Animal Research adopted by the CONCEA and the animals’ colony was submitted before the experiment to microbiological, serological and parasitological (search for protozoan, ectoparasites and endoparasites) tests, which confirmed the absence of pathogens, according to the Federation of European Laboratory Animal Science Associations (FELASA) guidelines on health monitoring; the experimental protocol was approved by the Ethics Committee for Animal Experimentation of Butantan Institute (CEUAIB n° 9416110219/ID 001708).

Housing conditions and experimentation with mice (*Mus musculus*) were also in strict accordance with the Ethical Principles in Animal Research adopted by the CONCEA and the animals’ colony was submitted before the experiment to microbiological, serological and parasitological (search for protozoan, ectoparasites and endoparasites) tests, which confirmed the absence of pathogens, according to the FELASA guidelines on health monitoring; the experimental protocol was approved by the Ethics Committee for Animal Experimentation of Butantan Institute (CEUAIB n° 9667040520).

### Permissive and non-permissive hosts for schistosomiasis – parasite exposure and sampling regimen

*S. mansoni* cercariae from a BH isolate (Parasitology Laboratory, Butantan Institute, Brazil) maintained by passage through golden hamsters (*Mesocricetus auratus*) and *Biomphalaria glabrata* snails were used for all infection and challenge experiments described here.

In our previous study^23^, 12 female rhesus macaques were exposed percutaneously to 700 cercariae on day zero and challenged with the same number of cercariae on the 42^nd^ week post-first infection. Plasma samples were collected from pre-infection, infection, and post-challenge at several weeks^23^ and were used in the present work. The selected time points and animals’ samples utilized in the present work will be described in the appropriate sections below. For macaques’ infection and for plasma collections, the macaques were anesthetized with xylazine (5 mg/kg body weight) and ketamine hydrochloride (10 mg/kg body weight). The experiment was terminated at week 20 post-challenge, the same time window as that when the macaques had eliminated the parasites from the primary infection phase^23^, by euthanizing the macaques with xylazine (5 mg/kg body weight) and ketamine hydrochloride (10 mg/kg body weight) sedation followed by a lethal dose of sodium thiopental (21 mg/kg body weight) and ketamine hydrochloride (36 mg/kg body weight).

Five golden Syrian hamsters (*Mesocricetus auratus*) aged 21 days-old were percutaneously infected with 100 cercariae, and serum samples were collected at weeks 0, 12, and 22 pi, as previously described^23^. Essentially, for hamsters’ infection and for serum collections, the hamsters were anesthetized with ketamine hydrochloride (10 mg/kg body weight) and xylazine (0.5 mg/kg body weight). The experiments were terminated at week 22 pi, when the parasites reached full maturity, by euthanizing the hamsters with ketamine hydrochloride (300 mg/kg body weight) and xylazine (60 mg/kg body weight).

Seven Balb/c mice aged 95 days-old were infected with 120 cercariae using the above-mentioned percutaneous method. For mice infection, they were anesthetized with ketamine hydrochloride (10 mg/kg body weight) and xylazine (0.5 mg/kg body weight). Serum samples were collected at weeks 0 and 6 pi with retro-orbital bleeding, after local anesthesia with proxymetacaine hydrochloride (5 mg/ml). The experiments were terminated at week 6 pi, when the parasites reached full maturity, by euthanizing the mice with ketamine hydrochloride (300 mg/kg body weight) and xylazine (60 mg/kg body weight).

### Design of the *S. mansoni* phage-displayed peptides library

A comprehensive synthetic DNA library with 119,747 sequences encoding 58-mer-long peptides was engineered by our group (Supplementary Information, Supplementary Table 4). The library covered all known 11,641 different *S. mansoni* proteins from V5.2 of the *S. mansoni* transcriptome expressed across the various parasite life-cycle stages. Each Open Reading Frame (ORF) was divided into 174-nt long fragments with a 21-nt overlap between consecutive segments, generating a total of 112,592 oligos, which were annotated with the respective Smp code, followed by a pipe symbol (|) and a three- to four-digit number (nnnn) representing the start nucleotide position of that given peptide within the full-length gene (ID: Smp_nnnnnn.n|nnnn). An additional 6497 putative alternatively spliced oligo sequences were designed in silico based on different combinations of short symmetric exon arrangements from 22 MEG family genes^62^ (ID: MEGnnnnnnnn|nnnn). In addition, 658 oligos encoding 255 newly identified genes^63^ were included (with variable ID names). Stop codons were removed, and synonymous mutations were introduced to optimize phage expression in *E. coli* and to eliminate restriction sites *NcoI, PstI*, and *EcoRV* from the 174-nt long sequences. Note that the oligo encoding the C-terminal end of each protein may have an overlap longer than 21-nt with the second-to-last oligo, to keep the 174-nt long insert size of the library design. Two 17-nt-long adapter sequences were added to each oligo at the 3’ and 5’ extremities, resulting in a total length of 208 nt. All oligonucleotide sequences are shown in Supplementary Table 4 (in Supplementary Information). The oligonucleotides were synthesized in duplicate on a releasable DNA microarray^25,26^ (Agilent Custom Oligo Library Synthesis OLS n° G7223A – 244k, 191-210 nt).

### Cloning *S. mansoni* phage-display library

We PCR-amplified the synthetic DNA library in 11 independent pools using the following conditions: synthetic DNA library (200 pM), 0.5 µM of each primer complementary to the 17-nt adapter sequences, 0.2 mM dNTPs, and 0.5 µL DNA Polymerase Invitrogen Platinum™ superFi per 50 µL reaction.

Forward Primer: 5’-CTGACCATGGCATCCAG-3’

Reverse Primer: 5’-TCATGCTGCAGACGATG-3’

The reactions thermal profile was
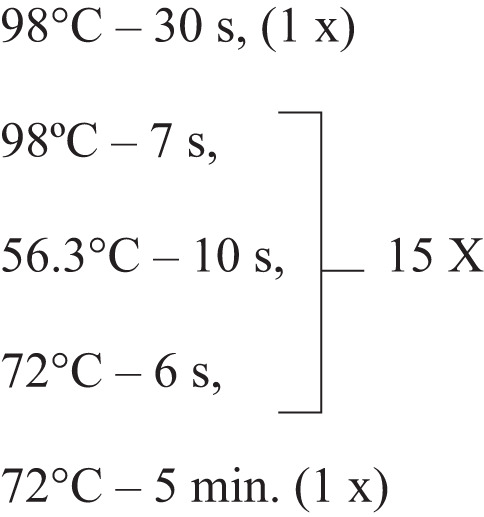


The PCR products from the 11 pools were combined, purified with Wizard SV Gel kit and PCR Clean-Up System (Promega) and quantified using Nanodrop and Qubit fluorometer. Amplicons (~2000 ng) and pG8SAET phagemid vector^64^ modified to include a *PstI* restriction site (~8000 ng) were separately digested with *NcoI* (High Fidelity version NcoI-HF) and *PstI* (High Fidelity version PstI-HF) restriction enzymes, following the manufacturer-suggested protocol. The resulting cohesive ends were ligated in 20 independent reactions at the inserts:vector proportion of 1:5 (NEB Instant Sticky-end Ligase Master Mix). The combined reactions were precipitated by adding 10% of 3 M sodium acetate (pH 5.2), 0.4% glycogen and 2.7-fold absolute ethanol. The mixture was incubated for 3 h at –80 °C. Subsequently, the tube was centrifuged at 12,000 x *g* at 4 °C for 30 min. The supernatant was discarded, and the tube was dried at 55°C. The resulting pellet was then resuspended in DEPC water.

A mass of 1000 ng from the resulting cloned library was amplified in *E. coli* TG1 strain and purified by QIAfilter Plasmid Purification Maxi kit (QIAGEN), using the manufacturer-suggested protocol. TG1 was electroporated with 5 µg of the plasmid library. The library at this step was titrated and presented ~800-fold representation. The library was amplified, packaged into pG8SAET bacteriophage by using helper phage M13KO7, and precipitated using the PEG/NaCl protocol, as described^64^. The phage-display library was amplified only once and applied to all samples in the following PhIP-Seq steps.

### Phage Immunoprecipitation-Sequencing (PhIP-Seq)

For this study, plasma samples from 10 rhesus macaques, which were not considered outliers in our previous analysis^23^, were selected (Rh2, Rh3, Rh4, Rh5, Rh6, Rh7, Rh8, Rh9, Rh11, and Rh12). As previously reported^23^, Rh1 was considered an outlier because an accidental leakage of liquid from the metal ring during infection resulted in this animal receiving a lower dose of cercariae at week 0; Rh10 was an outlier because this macaque became seriously unwell after Wk10 post-infection and had to be withdrawn from the study. Plasma collected after the *S. mansoni* infection at weeks 0, 8, 10, and 12 pi, and after the challenge at wk1pc and wk4pc were incubated with the phage-display library following the PhIP-Seq protocol as described^25,26^, with some adaptations. The tubes were previously blocked with 3% bovine serum albumin (BSA) in saline Tris-buffer with 0.5% tween-20 (TBST). Rhesus macaques’ IgGs were quantified using Monkey IgG ELISA Kit (Abcam- ab190549) and added at a final concentration of 2 μg/ml in 1 ml of the phage library, containing ~2 × 10^5^ copies of each sequence (5 × 10^10^ [PFU]/ml), diluted in Phage extraction buffer (20 mM Tris-HCl, pH 8.0, 100 mM NaCl, 6 mM MgSO4). All samples in duplicates were placed under rotation overnight at 4 °C. Phages that bound to IgG antibodies during the screening were immunoprecipitated (IP) using 40 μl of a mix containing proteins A and G coupled to magnetic Dynabeads (Invitrogen, 10001D, and 10003D) in the proportion 1:1. Reaction tubes were rotated again at 4 °C for 4 h. The IP reactions were placed in a magnetic rack, and beads were washed three times with 500 µl of Immunoprecipitation wash buffer (150 mM NaCl, 50 mM Tris-HCl, 0.1% NP-40, pH 7.5). IPs were resuspended in 30 µl of water and denatured at 90 °C for 10 min.

Immunoprecipitated DNA inserts were amplified by two rounds of PCR using Platinum superFi PCR Master Mix. In the first round, 56 µL reactions had 30 µL of PhIP and 0.1 µM of the 5’-GCAACACGatgaccatggcatccag-3’ and 5’-GCACCGGGTacgtactgcagacgatg-3’ primers, which contain part of the vector sequences (capital letters) and part of the adapters (lower letters) that flank the DNA insert of the library. The thermal profile followed the manufacturer 2-step PCR suggestions, and the denaturation and extension steps were repeated in 35 cycles. This reaction was purified using 1.2 X Agencourt AMPure XP, as suggested by the manufacturer.

The second PCR was performed to add Illumina adapters with 4 ng of DNA from the first PCR and a final concentration of 0.25 µM of the 5’-TCGTCGGCAGCGTCAGATGTGTATAAGAGACAGGCAACACGatgaccatggcatccag-3’ and 5’-GTCTCGTGGGCTCGGAGATGTGTATAAGAGACAGCACCGGGtacgt

actgcagacgat-3’ primers.

PCR cycles were modified to enhance specificity:
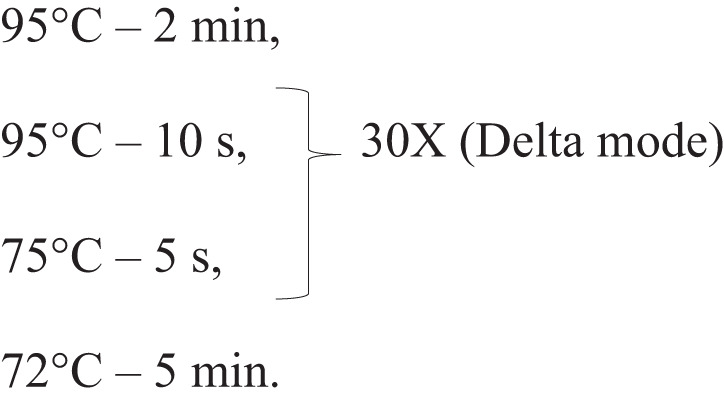


Delta mode decreases 0.5 °C and enhances 1 s at each cycle (Veriti 96 wells Thermal Cycler, Applied Biosystems). The second PCR product was purified with 0.65X Agencourt AMPure XP and paired-end-sequenced with Illumina HiSeqXTen at BGI.

Two replicas of the total library without going through precipitation steps were sequenced as an input control (~30 million reads per replicate). From the IP samples, 120 rhesus macaques’ PhIP-Seq samples (10 animals, 6 time points, 2 replicates = 120 samples), and 15 hamsters’ PhIP-Seq samples (5 animals, 3 time points per animal = 15 samples) were sequenced (~4 million reads per sample). As negative controls 12 immunoprecipitated phage samples that were incubated only with Protein A / Protein G magnetic beads, without plasma were sequenced (~2 million reads per sample).

### PhIP-Seq informatics and statistical analysis

The raw reads were processed using fastp (v 0.19.5)^65^ for sequences quality control and filtering, using default parameters. For mapping, each of the 119,747 oligonucleotides in the library (in the 174-nt insert stretch) was given a unique index, and then the reads were mapped against the library using Bowtie2 (v 2.3.4.3)^66^. Bowtie2 was used in *no-mixed* mode to disregard alignments in disagreement between paired-reads and *very-sensitive* mode, to increase alignment accuracy. As the DNA ORFs encoding the proteins were fragmented in silico into unique sequences in the library, there is no concern about splicing, and the sequencing reads must align completely with the sequence of one of the library fragments. Counting the number of reads mapped to each oligonucleotide in the library was done with Samtools (v 1.8)^67^ and the counts are shown in Supplementary Table 1 (in Supplementary Information).

We developed a PhIP-seq Bayesian statistical analysis to calculate the enrichment of each peptide based on the logODDs ratio, following the Aitchison logistic normal model^68^ (Supplementary Information). Our approach precludes the use of hard approximation (or convergence) methods such as the Markov Chain Monte Carlo (MCMC) algorithm. Instead, our sample is modelled as a large multinomial distribution, which gives as posterior a beta multivariate distribution with a known parameter, the sample frequency vector. The advantage is to use a log-odds transformation producing a multivariate normal distribution with digamma and trigamma functions as their means and variances – the Aitchison logistic normal distribution^68^. This is an exact method, not an asymptotic procedure, which is summarized in the Supplementary Information. Essentially, we used the non-informative prior, the simple multivariate beta distribution with unity vector parameter. The large sample size implies that the posterior is not significantly influenced by the choice of standard priors. The posterior probability function in this case can be understood as the average of the likelihood functions. Finally, taking a weighted average of the densities of a group of individuals, the meta-distribution of the group is obtained (Supplementary Information).

For this analysis, samples from the 10 rhesus macaques that were not considered outliers in our previous study^23^, were used (Rh2, Rh3, Rh4, Rh5, Rh6, Rh7, Rh8, Rh9, Rh11, and Rh12). The method of meta-analysis was used to compare groups, where each peptide represented a different meta-distribution, and the probability of the meta-distribution of one group being higher or lower compared with the other group was calculated to determine significant differences^69^; samples were divided into 3 groups: (1) two input libraries, (2) PhIP-Seq samples, and (3) negative controls. PhIP-Seq samples from the 10 rhesus macaques were further divided into six groups: wk0, wk8pi, wk10pi, wk12pi, wk1pc, and wk4pc. Three comparisons were made to identify enriched peptides: post-infection or post-challenge samples vs input libraries, post-infection or post-challenge samples vs negative controls, and post-infection or post-challenge samples vs wk0. Hamster samples were also divided into three groups, and similar analyses were performed to identify enriched peptides. The probability distribution curve of the post-infection or post-challenge group compared with each of the other three groups should be greater than or equal to 0.85 for the peptide to be considered enriched at a given time point.

### Analysis of proteins comprising enriched peptides

Genes encoding the proteins that comprise the enriched peptides were assigned to the different parasite life-cycle stages according to their published RNA-seq expression levels^29,30^. Data was downloaded with fasterq-dump (v.3.0.2) from PRJEB44842 for 12 egg samples^30^, integrity of data was checked using vdb-validate (v.3.0.2). Sequenced reads were filtered using fastp and aligned with STAR (v 2.7.3a) against the *S. mansoni* genome PRJEA36577 (v7) retrieved from WormBase (schistosoma_mansoni. PRJEA36577.WBPS14.genomicsoftmasked.fa). Read counting was performed with RSEM (v 1.3.1) using a previously published transcriptome annotation^29^. Data from other stages were downloaded from http://verjolab.usp.br/public/schMan/schMan3/macielEtAl2019/files/TPM_final.tsv^29^; only samples from whole worm were used, consisting of adult females (*n* = 37), adult males (*n* = 34), mixed adults (*n* = 20), juveniles (*n* = 9) and somula (*n* = 11). TPM values were used to calculate median values at each stage; each protein was assigned to the stage with the highest median expression (Supplementary Information, Supplementary Table 5). For each protein comprising an enriched peptide the subcellular location was predicted using the entire protein sequence and the Deeploc2 algorithm^70^ (Supplementary Information, Supplementary Table 5).

### Extracellular proteins analysis

Extracellular proteins assignment was done using single-cell data^31^, available online at http://verjolab.usp.br:8081/cluster-search/ (Supplementary Information, Supplementary Table 3).

SmAE protein was modeled using *Mus musculus* asparaginyl endopeptidase as a template (PDB 4NOK, 41.96% sequence identity), while SmCatB paralogs models were generated based on the crystal structure of *S. mansoni* cathepsin B1 (PDB 4I04) with sequence identities ranging from 85.71% to 95.60%. Homology models were generated using Swissmodel server (https://swissmodel.expasy.org/)^71–74^.

### Design and analysis of the peptide microarray

The peptide microarray was custom designed and ordered through PEPperPrint with 15-mer peptides printed on a glass slide in duplicate with a tiling offset of 2-aa, including a quality control HA tag (YPYDVPDYAG) (PEPperCHIP Custom Peptide Microarrays). Each array covered 23 peptide sequences from 7 different gut and esophageal gland proteins which were enriched in the rhesus macaques PhIP-Seq samples analysis (Supplementary Information, Supplementary Table 6). Four non-enriched sequences were added as negative controls (Supplementary Information, Supplementary Table 6). Rhesus macaques, hamsters and mice plasma/serum samples were individually screened with the microarrays using a 100-fold dilution following the manufacturer’s hybridization protocol. Secondary antibodies were 500-fold diluted (rhesus macaques – goat anti-Human IgG Fc Secondary Antibody, DyLight 650 (ThermoFisher); mice – Anti-Mouse IgG F(c), DyLight 649 conjugated (Rockland Immunochemicals); hamsters––Goat Anti-Syrian Hamster IgG H&L, DyLight 488 (ab102322)). The slides were scanned in the Agilent G2505C Sure-scan High-resolution technology scanner.

Sample selection: rhesus macaque (*n* = 8) plasma samples were collected at weeks 0 (pre-infection), 4, 6, 8, 10 post-infection (pi), 42 (challenge), weeks 1 and 4 post-challenge (wk1pc, and wk4pc). Here, we excluded samples from outliers (rhesus 1 and 10) and from medium responders to the infection (rhesus 7 and 12) based on a previous analysis^23^. Hamsters and mice serum samples (*n* = 5 and *n* = 7, respectively) were collected at pre-infection (wk0) and at the perfusion (wk6 and wk22, respectively).

Data analysis: The peptide microarray data were analyzed using the pepStat package from the Bioconductor project^75^. Innopsys MAPIX software outputs were stored in GPR file format and transformed into a log2 scale. A peptide z-scale was obtained to reduce batch/slide effects and nonspecific antibody binding signal. The *slidingMean* function was applied to check for neighboring peptides with 2-aa overlap sharing similar intensity values. Epitopes detected in at least 50% of samples with a normalized signal higher or equal to the median signal in the slide and FDR ≤ 1% were considered significantly reactive. Epitopes with 4-aa or longer were selected for further analyses.

### Dot Blot validation

Phage inserts encoding peptides Smp_075800.1|766, MEG8.21200110|154, MEG3.1710010|154, and MEG151001110|307 were individually amplified (specific primers – Supplementary Information, Supplementary Table 7), digested and cloned into the pG8SAET modified phagemid vector, as described above. Sequences were confirmed by Sanger sequencing and expressed into pG8SAET phage^64^. Additionally, pG8SAET without insert was used as WT negative control. Phages were titrated by plaque assay, and inserts were PCR confirmed. Dot blot was performed as described^76^ with modifications. For nitrocellulose membrane preparation, 5 μg of each phage (Pierce BCA Protein Assays) was suspended in 5 μl of TBS and dot blotted on each of 16 membrane strips. Membranes were air-dried for 1 hour at room temperature. Then, they were blocked for 2 hours at room temperature, followed by three 5-minute washes. Rhesus macaques’ plasma from eight animals was diluted 1000-fold in 1% BSA in TBS-T for wk0 or wk10pi samples and incubated with each of the 16 strips overnight at 4 °C. Then, membranes were washed and incubated with a 5000-fold dilution of Anti-human IgG conjugated with HRP (Sigma-Aldrich A8667) for 1 hour, followed by other wash steps. The reaction was revealed using Amersham ECL Prime Western Blotting Detection Reagent. Quantification was performed by scanning developed films and analysing the .tiff file with ImageJ software.

### Immunization of mice with *S. mansoni* phage-displayed peptides identified by rhesus macaque antibodies

Seven Balb/c female mice aged 35 days-old received an intraperitoneal immunization with a pool of 36 selected 58-mer peptides expressed as fusion proteins in the phage-capsid. We selected two different SmCatB peptides, two SmAE segments, 2 MEG-3.1 peptides, 14 MEG-4.1 peptides, 12 MEG-8.1 peptides, and 4 MEG-15 peptides (Supplementary Information, Supplementary Table 7). The DNA encoding each peptide was individually amplified under the specified conditions (Supplementary Information, Supplementary Table 7), cloned, checked by Sanger sequencing, and packaged into pG8SAET bacteriophage^64^. Mice were divided into immunized and control groups with seven animals each. Both groups received three doses with 15-day intervals of either WT phage (no-insert) or the pool of recombinant peptide phages (5 × 10^10^PFU/dose), diluted in PBS^77^. Serum was collected 28 days post-the-third immunization dose, and animals were infected 30 days after the last dose with 120 *S. mansoni* cercariae. At perfusion, wk6pi, adult worm load, eggs per mg of digested liver (5% KOH, at 37 °C, overnight), and weight were compared between the two groups. Sera from 28 days after the third dose from four immunized mice and WT control were selected based on the mean worm recovery per group to be screened in the peptide microarray and analyzed as described above.

### Reporting summary

Further information on research design is available in the Nature Research Reporting Summary linked to this article.

### Supplementary information

Supplemental Material

Reporting Summary

## Data Availability

The PhIP-Seq data generated in this study have been deposited in the NCBI Sequence Read Archive (SRA) under Accession number PRJNA998398. All other data supporting the findings of this study are available within the article and its Supporting Information files.
