# Supplementary material for: Schistosoma mansoni vaccine candidates identified by unbiased phage display screening in self-cured rhesus macaques
Source: NPJ Vaccines. 2024 Jan 4;9:5. doi: 10.1038/s41541-023-00803-x (PMC10767053; doi:10.1038/s41541-023-00803-x)
Supplement: Supplementary file 1 — Supplemental Material [file 41541_2023_803_MOESM1_ESM.pdf]

Schistosoma mansoni vaccine candidates identified by unbiased phage display screening in self cured rhesus macaques

Supplementary Information

Supplementary Tables 1 to 7 available at https://zenodo.org/record/8212883

PhIP-Seq statistical analyses comparison ……………………………………………….. 2

PhIP-Seq Bayesian statistical analyses ………………………………………………... 4

Supplementary Figures 4 to 7 …………………………………………………... 6 to 9

Supplementary References ……………………………………………………………... 9

PhIP-Seq statistical analyses comparison

We compared the Bayesian approach (described further below) to other two statistical approaches, namely: (i) Zero-inflated Generalized Poisson Distribution (ZIGP)[1] and (ii) Negative Binomial Distribution (NB)[2]. For each sample, the peptide count is normalized to reads per million (RPM), where the number of reads for each peptide is divided by the total reads mapped in the sample, then multiplied by 1×10^6. The peptides in normalized samples were grouped according to the number of counts in sliding windows with at least 30 different peptides and fitted to a chosen distribution (ZIGP or NB) and the distribution parameters were estimated using maximum likelihood.

For example, in a given sample, the phage insert counts of all immunoprecipitated peptides whose counts in the input equal to zero are grouped, forming a set with a certain distribution of these insert counts; then, the parameters are estimated to fit this distribution to the chosen distribution model. Once the group parameters are obtained, these values together with the counts in Reads Per Million (RPM) of the peptides that form this set are submitted to the density function of the chosen model to compute the p-values of the probability of enrichment of these peptides in this model. The same type of grouping is done with the other peptides that presented other count values in the input, so that for a given sample several distribution sets are obtained, each set having its estimated parameters and the corresponding peptides having their p-values of the probability of enrichment calculated. To determine the threshold for reproducibility between technical replicates, the values of log10 (−log10 (p-value)) in one replicate are grouped in sliding windows of width 0.05 in the range from the minimum and maximum log10 (−log10 (p-value)). Using all the peptides that are within each window, we calculate the median and the median absolute deviation of log10 (−log10 (p-value)) in the other replicate and plot them against the −log10 (p-value) for all windows.

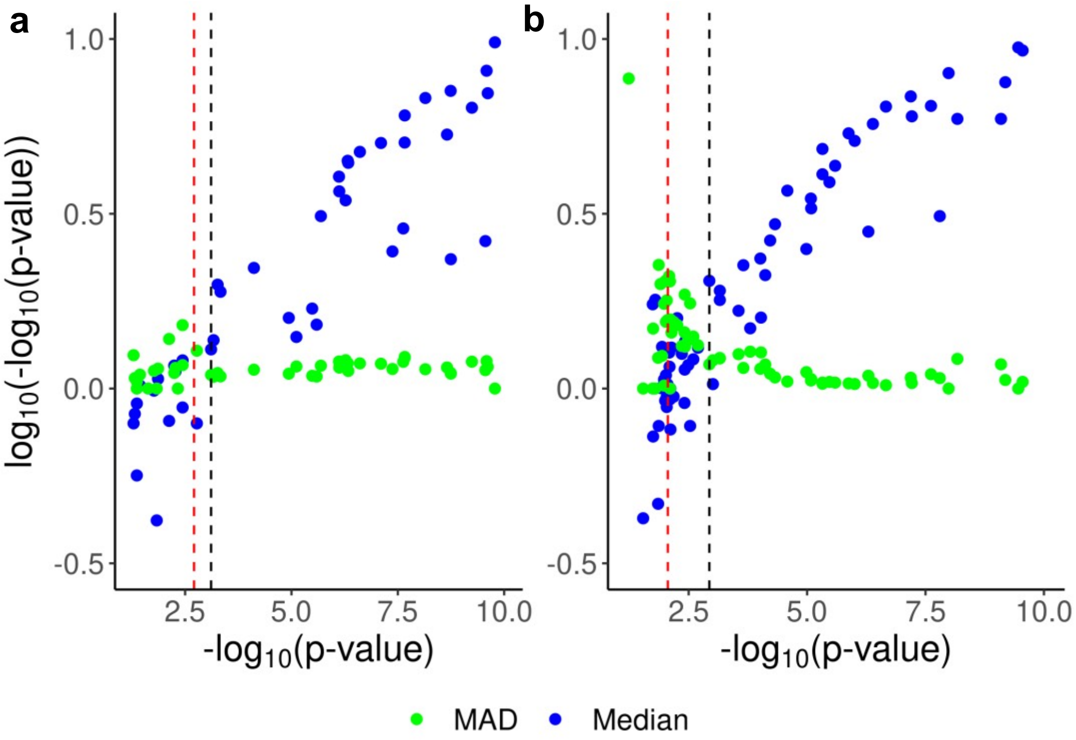

Supplementary Figure 1 – Determination of the reproducibility threshold between two replicates of each sample. The figure shows the example for sample wk0_Rh2. The reproducibility threshold is determined using the log10(-log10(p-value)) of the peptides from one of the replicates, grouped at 0.05 intervals between the rounded minimum and maximum values of the log10(-log10(p-values)), with a minimum of 30 peptides per group. Each group is displayed in the graph using the maximum value of -log10(p-value) of the peptides that make up the group; the median log10(-log10(p-value)) is plotted as a blue dot and its median absolute deviation (MAD) as a green dot on the graph. The dashed black line shows the threshold of reproducibility (TR) for this sample, determined by the maximum -log10(p-value) value of the first group in which the median of the log10(-log10(p-values)) (blue) is greater than the MAD (green). The average of the TR values of the set of samples of an experiment determines the threshold of significance (TS) (dashed red line) of the experiment, and only the peptides that have -log10(p-value)) above this TS value will be considered enriched. (**a**) Determination of threshold for p-values defined by the NB model; TS was calculated using all samples. (**b**) Determination of thresholds for p-values defined by the ZIGP model; TS was calculated using all samples.

The threshold for reproducibility (TR) in a given sample is defined as the point where the median is higher than the median absolute deviation value (Supplementary Figure 1, dashed black line). Then, the mean value of all calculated TR thresholds among all samples is used as the threshold of significance (TS) of enrichment in the experiment (Supplementary Figure 1, dashed red line). For the NB model the mean $-\log_{10}$(p-value) was 2.71 (Supplementary Figure 1a) and for the ZIGP it was 2.06 (Supplementary Figure 1b). Only peptides enriched in common in both analyses were used for further comparison with the Bayesian approach. To select enriched peptides in each rhesus macaques' group, it should not be enriched in 3 or more samples of negative controls nor in samples of rhesus macaques' wk0 and it should be enriched in at least 4 samples in a group (the set of rhesus macaque samples from a given week) for post-infection or post-challenge weeks. This resulted in several enriched peptides shown in Supplementary Table 8.

Supplementary Table 8 - Number of peptides enriched in common in the ZIGP and NB statistical analyses.

	wk8pi	wk10pi	wk12pi	wk1pc	wk4pc
Peptides	42	178	162	262	277
Related proteins	8	163	140	94	71

PhIP-Seq Bayesian statistical analyses

The Bayesian approach developed to perform the analysis was based on the logODDs ratio, following the Aitchison logistic normal model[3]. Each logODDs is represented by a normal distribution with mean and variance given by digamma and trigamma function, respectively.

$$\log ODDs \sim \theta$$

Where θ represents a normal distribution with mean and variance given by a digamma (F) and trigamma (ψ') function.

$$mean = \mathrm{F}\ (counts) - \mathrm{F}\ (library - counts)$$

$$variance = \sqrt[2]{\left((\psi'(counts)\right)^2 + \left((\psi'(library - counts)\right)^2}$$

Where *counts* represent the counts of a given peptide in a given sample and *library* represents the total counts of all peptides in a given sample.

For each peptide in each sample a distribution of logODDs is calculated (Supplementary Figure 2a), then in each group (the set of samples from the input, from negative controls or from a given week) the samples -distributions are summarized in a new meta-distribution for the group (Supplementary Figure 2b). Comparisons between groups are given by a probability distribution (P) measured by the area under the curve which did not overlap with any meta-distribution area of control groups (e.g. input, negative controls or wk0).

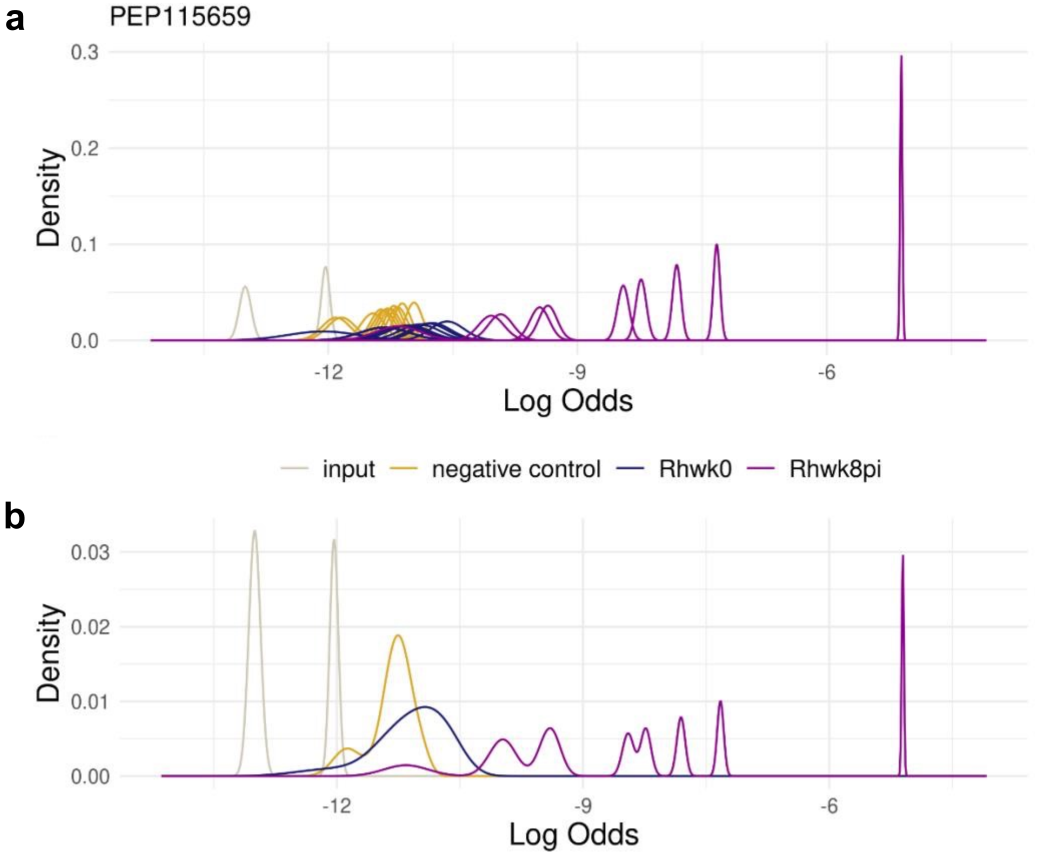

Supplementary Figure 2 – LogODDs distribution (a) for each sample and (b) for the group. The logODDs values are plotted in the x-axis and density is in the y-axis. Each group is represented by a different color: input, grey; negative controls, yellow; wk0, blue; and wk8pi, purple.

Using the Bayesian approach with P ≥ 0.85, the number of enriched peptides in all weeks is higher than in the NB/ZIGP analyses (Supplementary Figure 3).

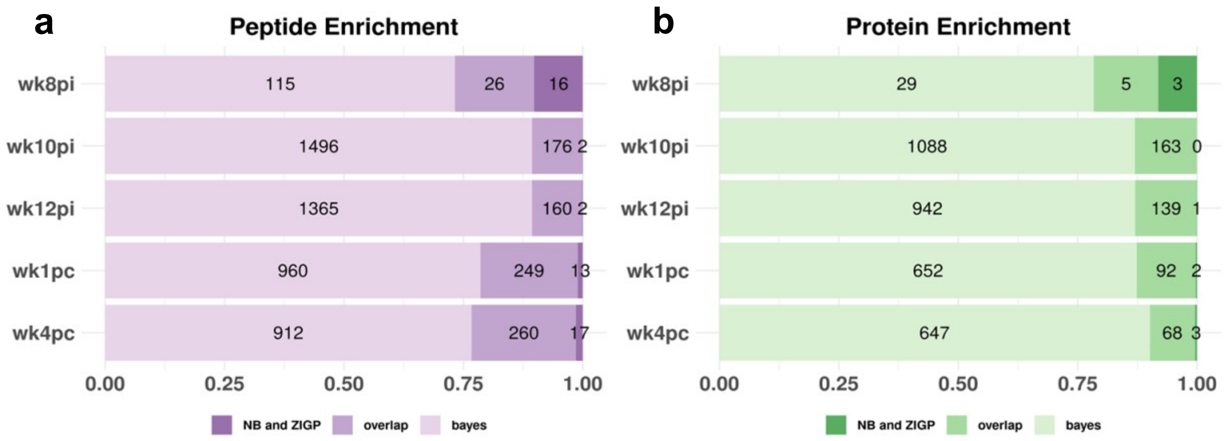

Supplementary Figure 3 – Barplot of (a) enriched peptides and (b) related proteins. Each experimental week is represented in the y-axis and the fractions of total number of peptides **(a)** or of related proteins **(b)** are represented in the x-axis. The number of enriched peptides/proteins are shown on the corresponding bar. Colors are scaled from light to dark, representing the enrichment in the Bayesian analysis, the overlap between the analyses, or the enrichment in the NB/ZIGP analysis; purple scale represents peptides and green scale represents the related proteins.

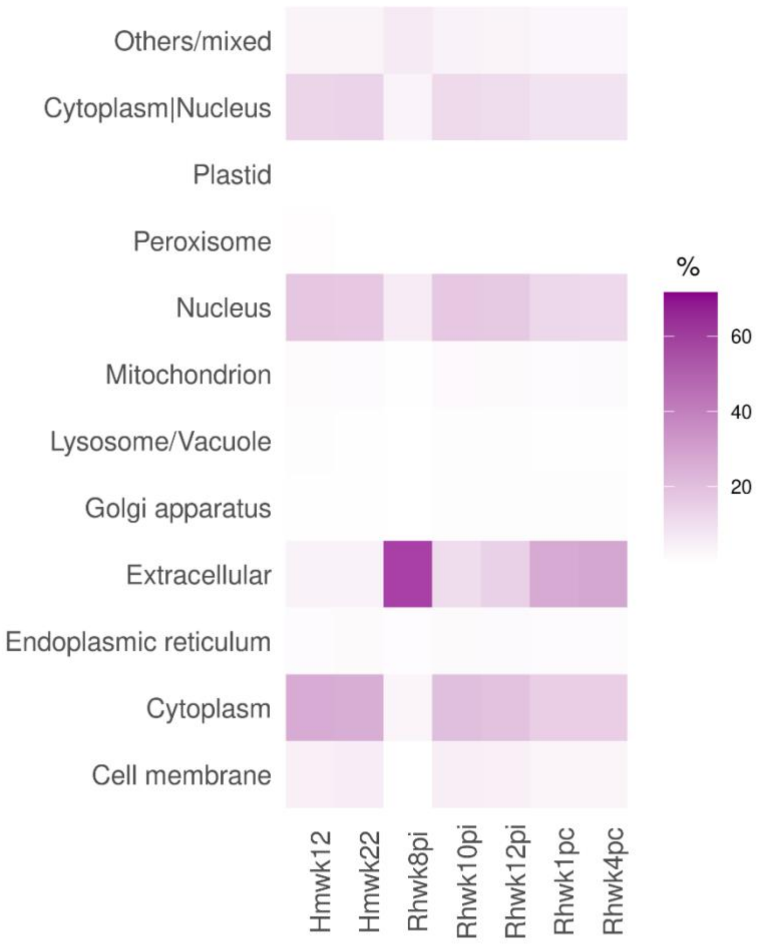

Supplementary Figure 4 - Heatmap displaying the percentage of enriched peptides comprising a specific subcellular localization compared to total peptides enriched at a given time point in samples IP with rhesus macaques or hamster serum. Proteins comprising the enriched peptides captured by rhesus macaques' and hamsters' antibodies were annotated using public data regarding *S. mansoni* life-cycle stage expression and subcellular localization. The percentage of enriched peptides in each subcellular location is shown in the scale at right.

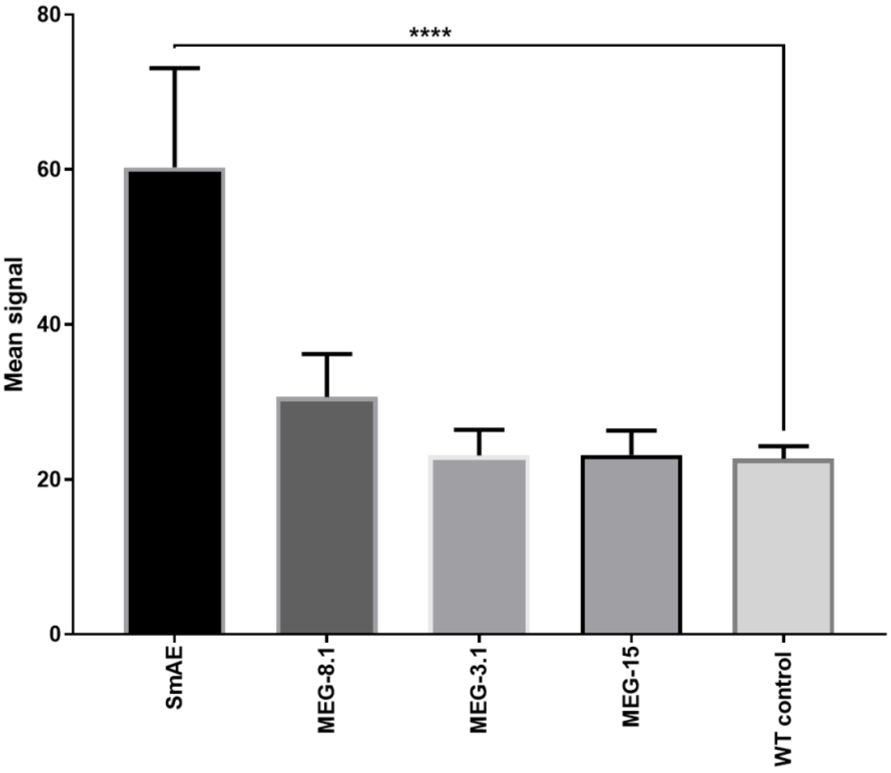

Supplementary Figure 5 – Mean chemiluminescent signal of dot blot assay of rhesus macaque's IgG antibodies against phages expressing four selected *Schistosoma mansoni* peptides. Four selected phages displaying peptide Smp_075800.1|766 *S. mansoni* Asparaginyl Endopeptidase (SmAE), peptide MEG8.21200110|154 (MEG-8.1), peptide MEG3.1710010|154 (MEG-3.1), or peptide MEG151001110|307 (MEG-15), and a wild-type-phage without insert (WT control) were dot blotted on each of sixteen nitrocellulose membrane strips. Each of 8 strips was blocked, washed, and incubated with plasma collected at week 10 post-infection (wk10pi) from each of the eight rhesus macaques, as described in Methods. Another 8 strips were similarly processed in parallel and incubated with plasma collected at week 0 (background). Membranes were developed with ECL chemiluminescence kit and images were quantified by ImageJ, as described in Methods. Mean signal among the eight macaques of each selected peptide incubated with wk10pi plasma (subtracted by its background signal, wk0) is shown on the y-axis. Whiskers are standard error of the mean (SEM). Mean signal for each peptide was compared with the mean signal against a wild-type phage with no insert (WT control). Statistical analysis was conducted using a two-way ANOVA. The significance level is denoted as follows: **** $P<0.0001$.

a

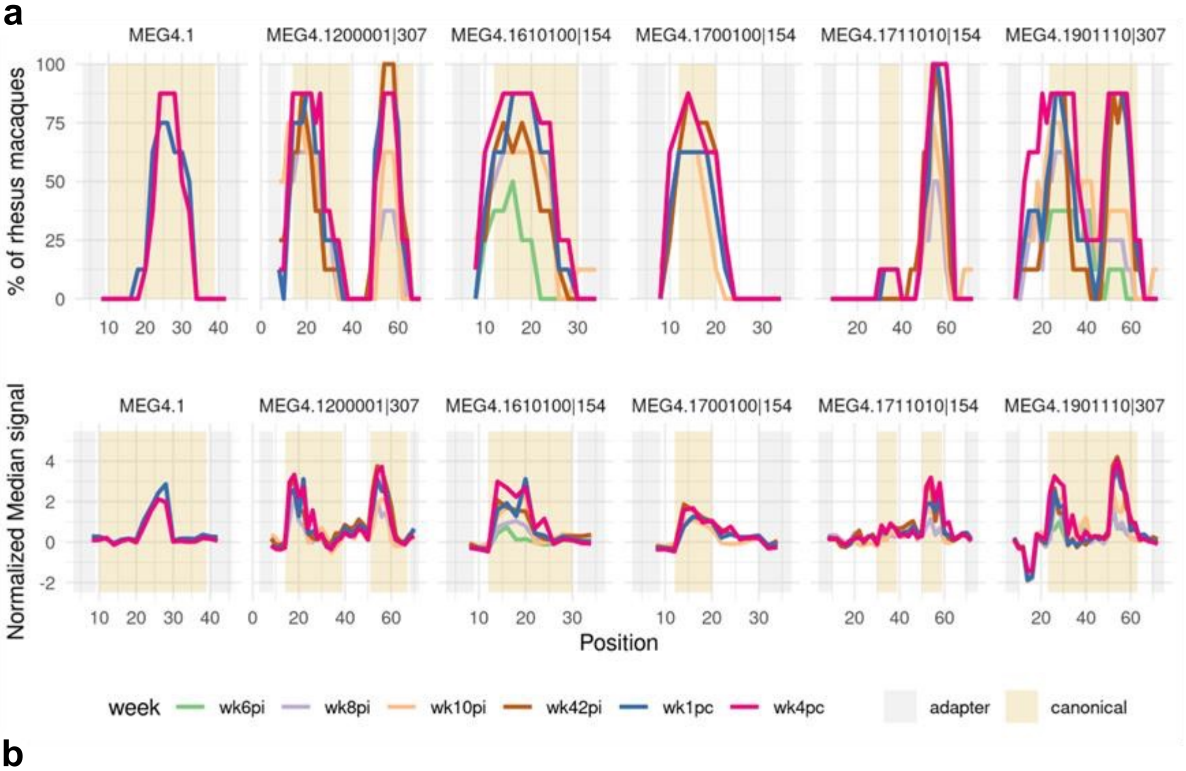

b

	ID	Epitopes
1	>MEG4.1	GSGSGSGNSMLKEYIEDNK**KDKHPTQK**TTPKPTTPKQ
2	>MEG4.1200001|307	GSGSGSGQHQR**QINDGTSDK**TSDTHTIKRTTPKPTTPKEQLPNLQHQR**QINDGTSDK**PKSIADIY
3	>MEG4.1610100|154	GSGSGSGQR**QINDGTSDK**TSDTHTIKRT
4	>MEG4.1700100|154	GSGSGSGQR**QINDGTSDK**EQLPNLQHQS
5	>MEG4.1711010|154	GSGSGSGNSMLKEYIEDKNVDIRIIENK**KDKHPTQK**EQLPNLQHQR**QINDGTSDK**TTPKPTTPKT
6	>MEG4.1901110|307	GSGSGSGIENK**KDKHPTQKQINDGTSDK**TSDTHTIKRTTPKPTTPK**QINDGTSDK**PKSIADFLNQ

Supplementary Figure 6 - *S. mansoni* MEG-4.1 epitope mapping with reactive plasma from rhesus macaques. (a) Peptide segments from MEG-4.1 (Smp_307220.2) (left panels) and five different peptide sequences containing different MEG-4.1 in silico-designed splice variants (IDs: MEG4.1nnnnnn|nnn) that were included in the peptide microarray; microarray adapter stretches at both ends of the sequences are highlighted with a grey background, while canonical positions have a salmon background. The upper charts represent the percentage of rhesus macaques (out of 8 animals) exhibiting antibody reactivity against epitopes from that peptide, and each line colour represents a different week (legends at the bottom of lower charts). The lower charts represent the median normalized signal intensities at each different week post-infection/challenge. **(b)** Table with peptide sequences from a canonical MEG4.1 stretch and five in silico-designed splice variants segments. The coloured sequence stretches highlight the epitopes KDKHPTQK (green) and QINDGTSDK (red) recognized by at least 50% of rhesus macaques (out of 8 animals).

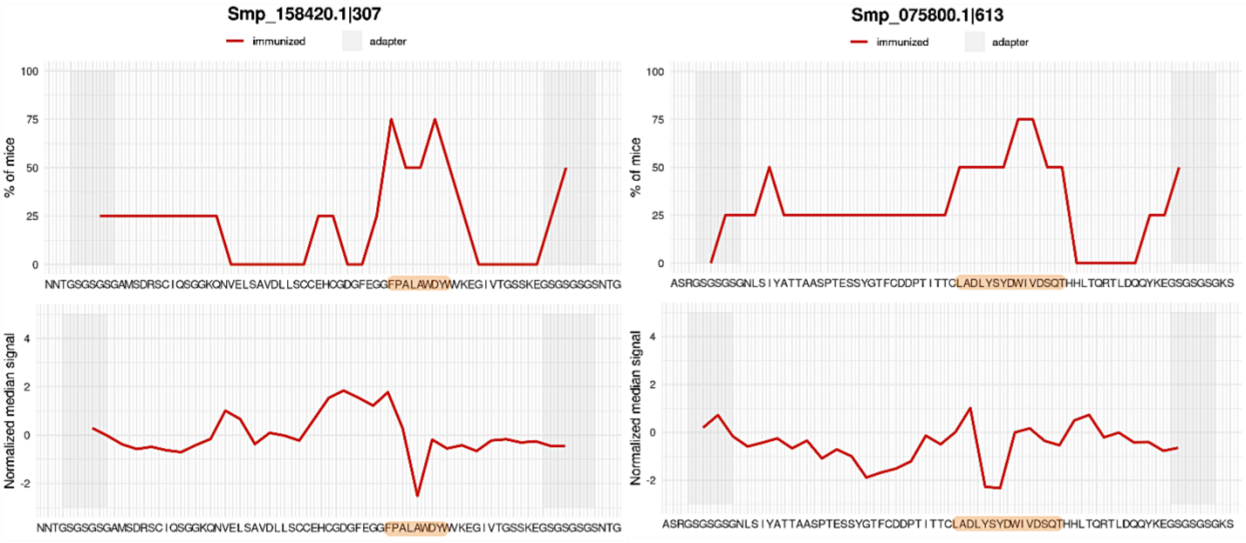

Supplementary Figure 7 – Epitope mapping with reactive serum from Balb/c mice immunized with a pool of peptides from *S. mansoni* blood-feeding and nutrient uptake proteins. Sera from four, out of seven immunized mice, was collected 28-days after the third immunization dose of phages encoding 58-mer peptides from SmCatB, SmAE, MEG-3.1, MEG-4.1, MEG-8.1, and MEG-15, and was used for screening with the peptide microarray. A significant antibody response was observed for epitopes within SmCatB (left charts: Smp_158420.1|307) and SmAE (right charts: Smp_075800.1|613) sequences. Each 58-mer phage-peptide sequence is shown at the bottom of each respective chart, and microarray adapter stretches at both ends of the sequences are highlighted with a grey background (legends at the top of upper charts). The upper charts represent the percentage of immunized mice (out of 4 animals) exhibiting antibody reactivity against epitopes from that peptide. The lower charts represent the median normalized signal intensity. The coloured block in each panel highlights the epitopes recognized by at least 50% of immunized mice (out of 4 animals). Images created with Biorender.com.

Supplementary References

1. Xu, G. J. *et al.* Comprehensive serological profiling of human populations using a synthetic human virome. *Science* **348**, (2015).
2. Lloyd-Smith, J. O. Maximum Likelihood Estimation of the Negative Binomial Dispersion Parameter for Highly Overdispersed Data, with Applications to Infectious Diseases. *PLoS ONE* **2**, e180 (2007).
3. Aitchison, J. The statistical analysis of compositional data. *Journal of the Royal Statistical Society: Series B (Methodological)* **44**, 139–160 (1982).
